# Corrigendum: Small genomic insertions form enhancers that misregulate oncogenes

**DOI:** 10.1038/ncomms15797

**Published:** 2017-06-01

**Authors:** Brian J. Abraham, Denes Hnisz, Abraham S. Weintraub, Nicholas Kwiatkowski, Charles H. Li, Zhaodong Li, Nina Weichert-Leahey, Sunniyat Rahman, Yu Liu, Julia Etchin, Benshang Li, Shuhong Shen, Tong Ihn Lee, Jinghui Zhang, A. Thomas Look, Marc R. Mansour, Richard A. Young

Nature Communications 8: Article number:14385 ; DOI: 10.1038/ncomms14385 (2017); Published: 02 09 2017; Updated: 06 01 2017

In the original version of Supplementary Data 1 associated with this Article, the list of predicted enhancer-associated insertions was inadvertently truncated. The HTML has now been updated to include the correct version of the Supplementary Data 1.

