# Supplementary material for: Small genomic insertions form enhancers that misregulate oncogenes
Source: Nat Commun. 2017 Feb 9;8:14385. doi: 10.1038/ncomms14385 (PMC5309821; doi:10.1038/ncomms14385)
Supplement: Supplementary Information — Supplementary Figures and Supplementary Tables [file ncomms14385-s1.pdf]

Supplementary Figure 1

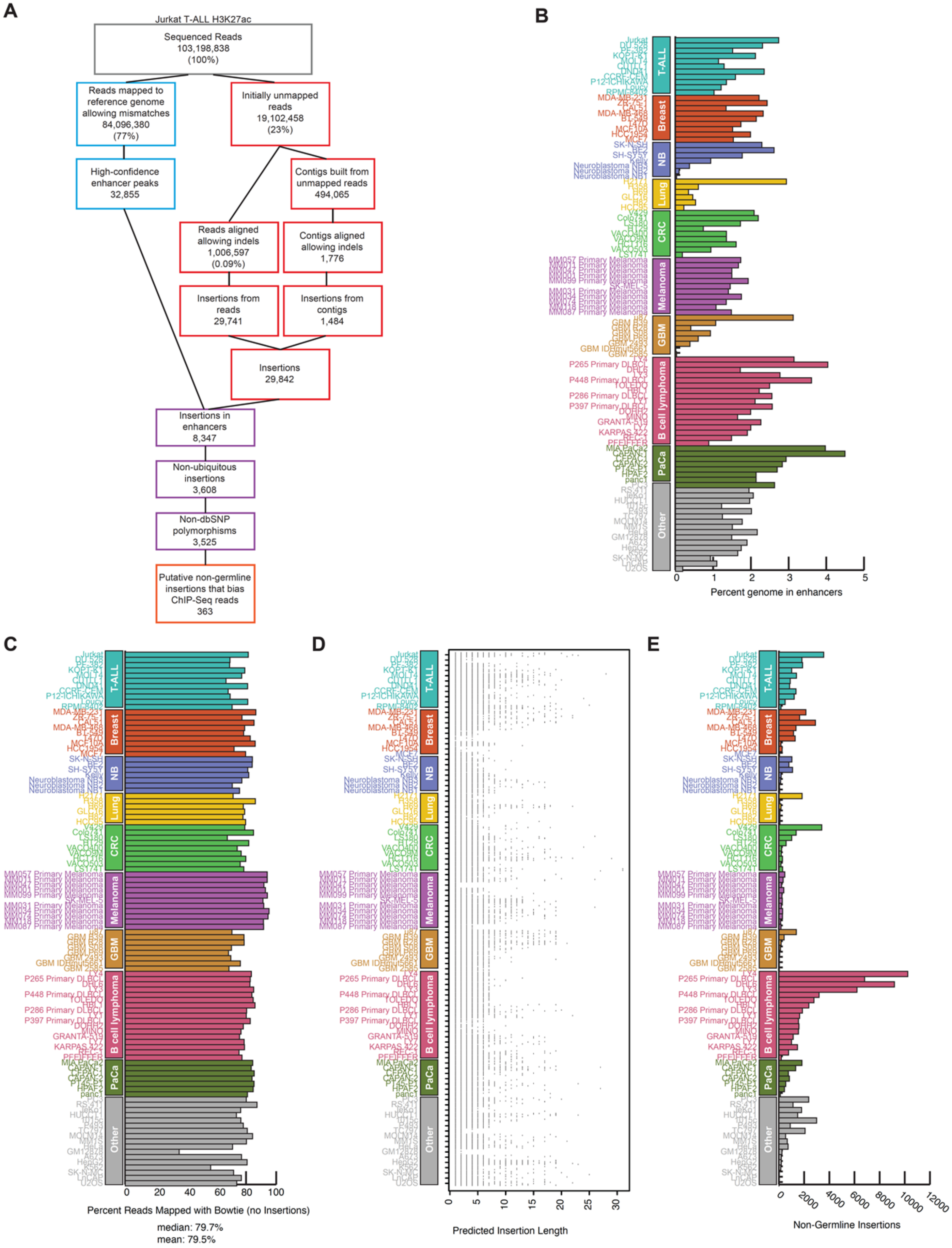

Supplementary Fig. 1: A computational pipeline to predict enhancer-associated insertions

A) A computational pipeline for identification of insertions from ChIP-Seq data. The majority of ChIP-Seq reads in Jurkat cells map to the reference genome using bowtie without accounting for insertions. Less than 0.1% of initially unmapped reads successfully map when allowing for insertions. Initially discarded reads are assembled into 1,776 contigs and are aligned to the reference genome. After processing, 29,842 insertions are detected relative to the reference genome using H3K27ac ChIP-Seq data, of which 8,347 are contained within an H3K27ac-enriched region. 3,608 enhancer-associated insertions are not predicted in 3 or more samples, and 3,525 enhancer-associated insertions are not in dbSNP, which suggests they are not likely to represent germline variation. 363 non-germline enhancer-associated insertions are predicted to alter enhancer activity.

B) Per-sample percentages of genome (presuming 3,400,000,000-nucleotide genome) contained in H3K27ac-enriched regions, which represent putative active enhancers.

C) The majority of reads in each H3K27ac ChIP-Seq sample mapped to the reference genome with bowtie, which does not account for insertions.

D) Dotplot of the sizes of predicted insertions in each tested sample.

E) Per-sample counts of insertions that are not thought to be germline variation based on recurrence across samples and presence in dbSNP.

Supplementary Figure 2

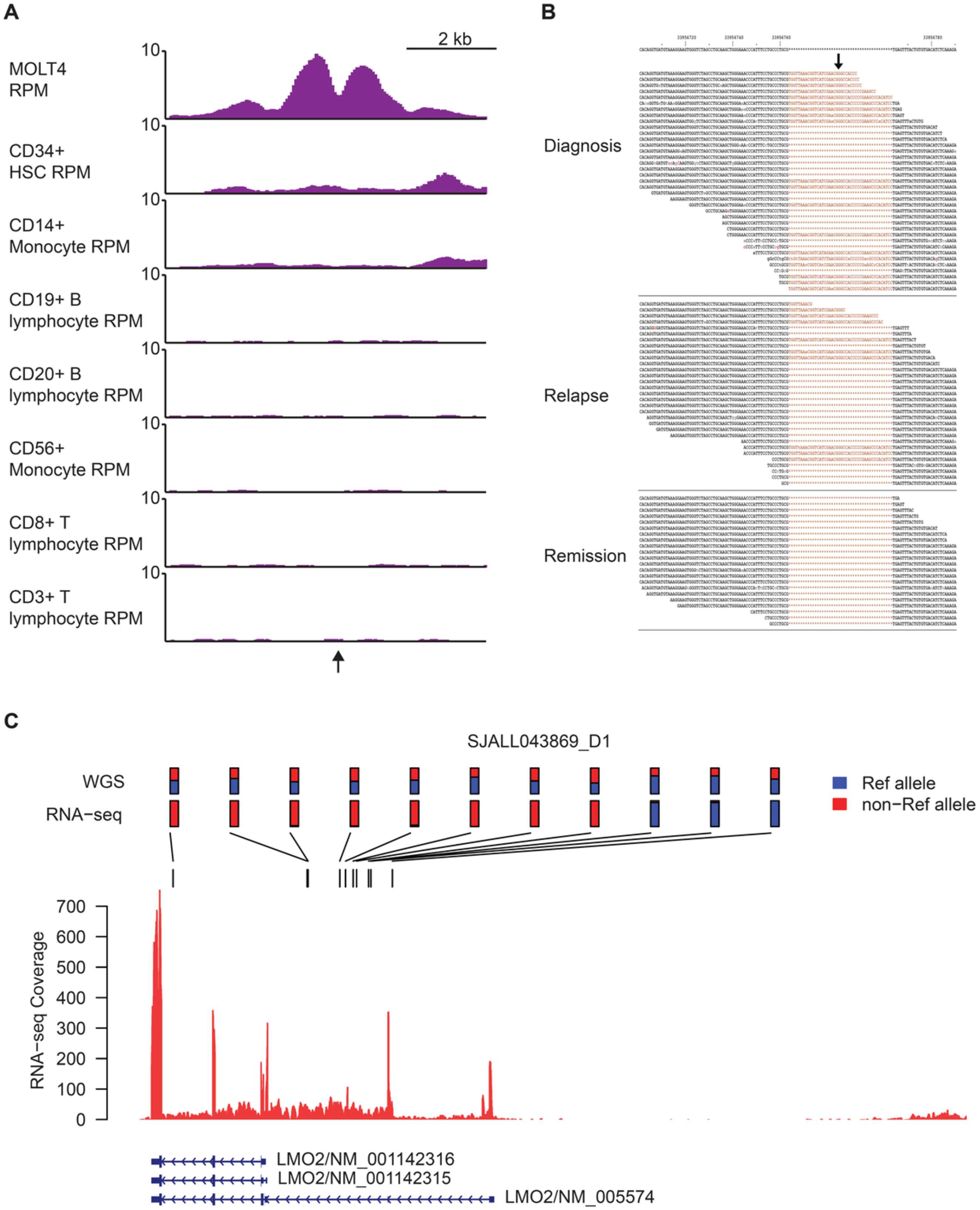

Supplementary Fig. 2: A confirmed insertion near *LMO2*

A) H3K27ac ChIP-seq signal at the *LMO2*-proximal insertion does not suggest that the mutation in MOLT4 T-ALL cells reactivates an enhancer active at some other stage of blood development. ChIP-seq read counts from human blood samples at the insertion site are displayed in purple.

B) Whole-genome sequencing reads from diagnosis (top), relapse (middle) and remission (bottom) DNA of the same T-ALL patient with a 45-bp somatic insertion located 42.9kb upstream of the *LMO2* transcription start site (location indicated by arrow). The location of the somatic insertion (chr11:33956762 in hg19) matches that of an 8-bp insertion identified in MOLT4 cell line. The inserted sequence (shown in brown) contains a MYB-binding site.

C) *LMO2* is expressed from one allele in a patient with an *LMO2*-proximal insertion. Top: Whole-genome sequencing coverage and RNA-seq read coverage of 11 germline SNPs with heterozygous genotypes (VAF ranges 0.3-0.7, top). Whole-genome sequencing coverage shows both alleles are present in similar ratios. RNA-seq shows a bias toward one allele. The locations of the interrogated SNPs are noted in black. The location of somatic insertion is marked by an arrow.

Supplementary Table 1:
ChIP-Seq datasets used

Sample	GEO H3K27ac	GEO Input	GEO Project	Pubmed Citation
TC797	GSM1820938	GSM1820932	GSE70870	26220994
A673	GSM1517563	GSM1517568	GSE61944	25453903
BE2	GSM1680101	GSM1680103	GSE65664	26560027
BT 549	GSM1589476	GSM1589475	GSE65201	26406377
CAL51	GSM1693015	GSM1693027	GSE69112	26051943
CAPAN1	GSM1574235	GSM1574271	GSE64560	26769127
CAPAN2	GSM1574236	N/A	GSE64560	26769127
CCRFCEM	GSM2037781*	GSM2311757*	NEW HERE	N/A
CFPAC1	GSM1574237	N/A	GSE64560	26769127
Colo741	GSM1296640	N/A	GSE53602	N/A
CUTLL1	GSM1252938	N/A	GSE51800	24374627
DHL6	GSM1254194	GSM1254195	GSE51800	24374627
DND41	GSM1003462	GSM1003558	GSE29611	22955616
DOHH2	GSM1703895	GSM1703897	GSE69558	26229090
DU528	GSM2037785*	GSM2311758*	NEW HERE	N/A
GBM_2493	GSM1866060	GSM1866061	GSE72468	26455392
GBM_2585	GSM1866062	GSM1866063	GSE72468	26455392
GBM_B39	GSM1866064	GSM1866065	GSE72468	26455392
GBM_IDHmut5661	GSM1866066	GSM1866067	GSE72468	26455392
GBM_P69	GSM1866068	GSM1866069	GSE72468	26455392
GBM_R28	GSM1866070	GSM1866071	GSE72468	26455392
GBM_S08	GSM1866072	GSM1866073	GSE72468	26455392
GLC16	GSM1526704	GSM1526701	GSE62614	25490451
GM12878	GSM733771-SRR227633	GSM733742	GSE29611	22955616
GRANTA_519	GSM1703901	GSM1703902	GSE69558	26229090
H2171	GSM894067	GSM1008615	GSE36354	23021215
H358	GSM1635575	N/A	GSE66992	26656844
H69	GSM1526703	GSM1526705	GSE62614	25490451
H82	GSM1526706	GSM1526702	GSE62614	25490451
HBL1	GSM1254196	GSM1254197	GSE51800	24374627
HCC1954	GSM721136	GSM721139	GSE29069	22156296
HCC95	GSM1635578	N/A	GSE66992	26656844
HCT116	GSM945853-SRR504923	GSM749774	GSE31755	N/A
HeLa	GSM733684	GSM733659	GSE29611	22955616
HepG2	GSM733743	GSM733732	GSE29611	22955616
HPAF2	GSM1574238	N/A	GSE64560	26769127
HT29	GSM1296643	GSM1296642	GSE53602	N/A
HUCCT1	GSM1670702	N/A	GSE62275	26439301
JEKO1	GSM1703903	GSM1703904	GSE69558	26229090
Jurkat	GSM1296384	GSM1296386	GSE50622	25043025
K562	GSM733656	GSM733780	GSE29611	22955616
KARPAS_422	GSM1703909	GSM1703910	GSE69558	26229090
Kelly	GSM1532401	GSM1532403	GSE62725	25416950
KOPTK1	GSM2318734	GSM2311759*	NEW HERE	N/A

LnCAP	GSM686937	GSM686947	GSE27823	21572438
Loucy	GSM2037788*	GSM2311760*	NEW HERE	N/A
LS174T	GSM1365900	GSM1197323	GSE49320	24952462
LS180	GSM1890754	GSM1890756	GSE73319	26752646
Ly1	GSM1133646	GSM1133654	GSE46663	24332044
LY3	GSM1254198	GSM1254199	GSE51800	24374627
LY4	GSM1254200	GSM1254201	GSE51800	24374627
MCF10A	GSM1829629	N/A	GSE71196	26657629
MCF7	GSM945854	GSM945859	GSE31755	N/A
MDA MB 231	GSM1204474	GSM1204476	GSE49651	24885402
MDA MB 468	GSM1589470	GSM1589471	GSE65201	26406377
MIAPaca2	GSM1574239	GSM1574272	GSE64560	26769127
MINO	GSM1703912	GSM1703913	GSE69558	26229090
MM001 Primary Melanoma	GSM1484294	N/A	GSE60663	25865119
MM011 Primary Melanoma	GSM1484295	N/A	GSE60663	25865119
MM031 Primary Melanoma	GSM1484296	N/A	GSE60663	25865119
MM034 Primary Melanoma	GSM1484297	N/A	GSE60663	25865119
MM047 Primary Melanoma	GSM1484298	N/A	GSE60663	25865119
MM057 Primary Melanoma	GSM1484299	N/A	GSE60663	25865119
MM074 Primary Melanoma	GSM1484300	N/A	GSE60663	25865119
MM087 Primary Melanoma	GSM1484301	N/A	GSE60663	25865119
MM099 Primary Melanoma	GSM1484302	N/A	GSE60663	25865119
MM118 Primary Melanoma	GSM1484303	N/A	GSE60663	25865119
MM1S	GSM894083	GSM894087	GSE36354	23021215
MOLM14	GSM1587893	GSM1587891	GSE65138	26416749
MOLT4	GSM2037790*	GSM2311761*	NEW HERE	N/A
Neuroblastoma NB1	GSM1532414	N/A	GSE62725	25416950
Neuroblastoma NB2	GSM1532415	GSM1532416	GSE62725	25416950
Neuroblastoma NB3	GSM1532417	GSM1532418	GSE62725	25416950
NMC_1015	GSM1820955	GSM1820946	GSE70870	26220994
OCI_LY7	GSM1703918	GSM1703919	GSE69558	26229090
P12 ICHIKAWA	GSM2037794*	GSM2311762*	NEW HERE	N/A
P265 Primary DLBCL	GSM1254210	GSM1254211	GSE51800	24374627
P286 Primary DLBCL	GSM1254212	GSM1254213	GSE51800	24374627
P397 Primary DLBCL	GSM1254202	GSM1254215	GSE51800	24374627
P448 Primary DLBCL	GSM1254216	GSM1254217	GSE51800	24374627
P493	GSM1036405	GSM1036408	GSE42262	N/A
panc1	GSM818826-SRR353689	GSM818828	GSE31755	N/A
PC3	GSM1383871	GSM1383876	GSE57498	24916973
PF382	GSM2037796*	GSM2311763*	NEW HERE	N/A
PFEIFFER	GSM1703923	GSM1703924	GSE69558	26229090
PT45P1	GSM1574241	N/A	GSE64557	26769127
REC1	GSM1703925	GSM1703926	GSE69558	26229090
RPMI-8402	GSM1442003	GSM957615	GSE59657	25394790
RS411	GSM1841292	GSM1841288	GSE71616	N/A
SHSY5Y	GSM1602665	GSM1602669	GSE65664	26560027
SKMEL5	GSM1484304	N/A	GSE60666	25865119
SKMNC	GSM1517538	GSM1517543	GSE61944	25453903

SKNSH	GSM2037798*	GSM2311764*	NEW HERE	N/A
T47D	GSM1589474	GSM1589473	GSE65201	26406377
TOLEDO	GSM1254202	GSM1254203	GSE51800	24374627
U2OS	GSM1356567	GSM1231607	GSE44672	25043018
u87	GSM894065	GSM894097	GSE36354	23021215
V429	GSM883681	GSM883667	GSE36204	22499810
VACO 400	GSM883684	GSM883666	GSE36204	22499810
VACO 503	GSM883682	GSM883671	GSE36204	22499810
VACO 9M	GSM883683	GSM883674	GSE36204	22499810
ZR 75 1	GSM1589472	GSM1589477	GSE65201	26406377

DISPLAY ONLY

Jurkat MYB mp	GSM1442005			
MOLT4 MYB	GSM1519643			
MOLT4 TAL1	GSM1519646			
CD34 H3K27ac	GSM772894			
CD14 H3K27ac	GSM1003559			
CD19 H3K27ac	GSM1027287			
CD20 H3K27ac	GSM1003459			
CD56 H3K27ac	GSM1027288			
CD8 H3K27ac	GSM1102781			
CD3 H3K27ac	GSM1058764			
Jurkat CTCF	GSM1689152	GSM1689151	GSE68976	26940867

Supplementary Table 2:
Insertions tested with high-throughput sequencing of pooled PCR products

ID	PREDICTED INSERTION	REF PRESENT	INS PRESENT	FWD PRIMER	REV PRIMER
chr11_124814827_124814827_A	A	TRUE	TRUE	GGATCAATCACTGTAGCAAGGAG	AGGGTTTGTTTGTGTTTTGTGAG
chr16_49476019_49476019_T	T	TRUE	FALSE	CCACAATTCTAAAACCCTTTTCA	GTTGTACGGACTTGCAGATTTTC
chr3_59742226_59742226_CACT	CACT	FALSE	TRUE	AAAGGTAGTCAATACCCCAGGAG	TAGTTACCAAATGCAAGCCAAGT
chr5_62860132_62860132_A	A	TRUE	TRUE	GGCTCCACAGCTTCACTTTAGTA	CCTAGCCTTCCACTGTCCTTAAT
chr6_25049622_25049622_A	A	TRUE	TRUE	GACACTTCTCGAAAGAAGGCATA	TGAGAAATCTCCAAACTGCTTTC
chr11_77912884_77912884_C	C	TRUE	TRUE	CTTCTGATGCTCACAAGAAACCT	CTGTGCTGCAGATGTAAAAGTCA
chr2_7019806_7019806_T	T	TRUE	TRUE	GCAGTTGGTACAAATATGTCTTGC	GGCTTTCTGAATGAAAGTCGTAA
chr4_109387724_109387724_T	T	TRUE	TRUE	CTCCCTCCCACTAGTACTGACCT	CCCTGCCTTTGAAGAACTAAGAT
chr10_102295437_102295437_T	T	TRUE	TRUE	TCAGCCTTTGCTCTACATCTACC	AGCTCTACATCCTAGGCTCCACT
chr1_221916195_221916195_T	T	TRUE	FALSE	GAGGTGACTTGACAAACGCTAAG	AGAGGGGCAGCGACATCT
chr15_27910479_27910479_A	A	TRUE	TRUE	GAGCAGACAGTAGGTCAGCATTT	TGTGGAAGCAAATAATTTTACCG
chr17_65449431_65449431_T	T	TRUE	FALSE	CAGGGTACATGGAAAATGCTTAG	TTTTCTCCATTACCTCTCACCAA
chr17_76816557_76816557_A	A	TRUE	TRUE	AACATAGCAAGACCCCTGTCTCT	AAAGTGACTGATTCATGGCAGAC
chr2_106513552_106513552_TT	TT	TRUE	TRUE	CCAACTAGTGTCCAGAAAACCTG	TCCGTACAACAAATCCCTATGAC
chr2_232528615_232528615_T	T	TRUE	FALSE	AGCCTAGTCCCCTCTGATTTTT	CATGACTGGAGAGTACCCGTAAC
chr3_6584125_6584125_T	T	TRUE	TRUE	CTCAAGTAGCTGGGACAACAGAG	AGTTTGAGAAGCATGAAAAGCAC
chr19_13275283_13275283_C	C	TRUE	FALSE	GAGGCAGAGGATCCAGTTTTACT	AGTGTGCACAGGAAAAGGAAAC
chr4_40057698_40057698_T	T	TRUE	FALSE	AGTCGCCTACCCCTTTGTCT	AAAAACAAAACAAACACAAGGTC
chr5_142181175_142181175_G	G	TRUE	TRUE	GACAAACATAAAAACCCCAATCA	AGGAAAACAGTTGGGTACAGTGA
chr14_64856429_64856429_A	A	TRUE	TRUE	GCATCTGTAATCCCAGCTACTTG	GCCACTGGAAAAGGAAGTAAGTT
chr22_29207152_29207152_T	T	TRUE	TRUE	CTCTTTGGCATCTACAAAATGCT	AAAGCTTGCTTCTCTTGCTACCT
chr10_2804386_2804386_TC	TC	TRUE	TRUE	GTGTGAAAGAAATCTCCGGAAC	TAGACCAGTGGCTATCGAAATGT
chr11_36429964_36429964_AGG	AGG	TRUE	TRUE	CCAATGTTGTCCCATGTAAAAGT	TTCAAAACAAGAAGACTCCCAGA
chr1_202135424_202135424_G	G	FALSE	TRUE	CTCATCCTCAGCTGTCTGTAGGT	CTCTGAGGAGCAATTCAAGTACC
chr12_125172967_125172967_A	A	FALSE	FALSE	CAAAGCCAGCAATAGCTCTGTAT	TTCCCATTGGAATGAGTTATGTC
chr13_25779970_25779970_T	T	FALSE	TRUE	ACTGCATAGCTTTGCTTCACTTC	ATTGGACAACACCTGAGTGAGAC
chr14_107283384_107283384_A	A	FALSE	TRUE	TTCCTGACCCAGTATGAGAAAGA	TCATTCCCAAAATAACACATTCA
chr15_33416754_33416754_GA	GA	FALSE	TRUE	TCTCCCAAAGTGATCCTAATGTG	GGAGCACAAAGGAGTGCTATTTA
chr15_98651202_98651202_T	T	FALSE	TRUE	TGCCAAGAAGTGTACAGAGAGGT	GGGTAAATACTCCTTTTCCCAAA
chr17_15014341_15014341_CTT	CTT	FALSE	TRUE	GTGTCTCCAATTGCACAGTAACA	TTTAAACTGCCAGCAAAAATATGA
chr17_54887265_54887265_C	C	TRUE	TRUE	ACACCCCTCTACACACATACACC	AAGGGTCTGTGCAAGGATTGT
chr20_31580894_31580894_A	A	TRUE	TRUE	TGAAAAACTATTTGGCAGCATCT	CCTTTGGGCCTAAGAAATGTTAT
chr20_31582392_31582392_AA	AA	TRUE	TRUE	GGAAAAGATGGACAACATAGGTG	TTTTTAGTGGAGACAGGGTTTCA
chr21_34915849_34915849_T	T	TRUE	TRUE	CCTGTTTCAGAACCGAGTTAAGAT	TGAAGCTCGCAATAATAAAGAGG
chr2_202123719_202123719_AGTT	AGTT	FALSE	TRUE	AAGGGTTTCCTTTTATCTCTCCA	TATCTCCTTCAAGCCTCACAGTC
chr22_26977304_26977304_G	G	TRUE	TRUE	TAACTCCTACTGGCATCATTTGC	TTCTTTCTCAATCCATCCTTCTG
chr3_131664048_131664048_CTTAT	CTTAT	TRUE	TRUE	AGTTCCAAAAATTGGGCATAGAT	TTTGTTACCAGGTTTTGAGAGGA
chr3_53134986_53134986_A	A	TRUE	TRUE	AACTTTCTGGGGAAATGGAGATA	CATAAACCCTGAATGAGTTCCAG
chr4_156504660_156504660_A	A	TRUE	TRUE	GCATGTCAGGCTTTTGTTTATTT	GAAGCCAAAGTTTTCTCTTCTCC
chr4_164465863_164465863_A	A	TRUE	TRUE	CAGAAATGCAGAAGTAACCAACC	TGGTGGTAAAACAGTGAGAAACA
chr4_178362311_178362311_CACTTCAAGCAT	CACTTCAAGCAT	FALSE	TRUE	TCACGACCCTCATTTTAAAGAAA	GCCTGTATTGAAAGAGGGAACTT

chr6_14243688_14243688_AA	AA	FALSE	TRUE	ACAAAGAAGGAAACGAGACACTG	TGTCACAGAGATGGTTGATGAAG
chr6_36913923_36913923_AA	AA	FALSE	FALSE	GAGAAGCAAATGCCAGAGATG	GAGGAGAGAGAGGAATGGAAGAG
chr7_158989830_158989830_G	G	FALSE	TRUE	GACGTAATACCCTGAGTCACCTG	CTAAAGGACCAGAGGAAGTCAGC
chr8_135333543_135333543_A	A	TRUE	TRUE	GGGTTTTCATAGTTTTGGGTTTT	TTGGCCTTTATATGACTTTTCCA
chr8_23525724_23525724_T	T	TRUE	FALSE	TTAGGCACCAGAATTCTTCTCAG	TTCATCTGCTAATTCCTCCAATC
chr1_108626440_108626440_A	A	TRUE	FALSE	CTGTGCCTTTAGGAAAGAAGACA	CTCCTGTTTCAGAGGAGATGCTA
chr11_106448087_106448087_G	G	TRUE	FALSE	TTCCTTCAATATTTCTCCCTTCA	AAATGAGAAGGAACCAGAAAACC
chr11_78062377_78062377_G	G	TRUE	FALSE	CTTCTGTTTCACATGTCATAGCC	CTGATGAAAGGGATGACAGTAGC
chr11_9595135_9595135_C	C	TRUE	FALSE	ATAGGCCCGGAACACCAC	GTCTCCTCAGGTCCAGTCTCAG
chr1_229832162_229832162_G	G	TRUE	TRUE	GCTTTAGAAACGCTGTTTTGTGT	AGGCATTCTAGATTGGTTGGTTT
chr13_99910301_99910301_G	G	TRUE	FALSE	ATAGAGACGGGGTCTCACTATGG	CAGAAGTGGAAGTGCTGAAAAAT
chr14_104215134_104215134_A	A	TRUE	FALSE	TACAGAGGGTGGTAAAGGGGTAT	AATGGTTCCAGTCTTTTCCCTAA
chr14_92340403_92340403_TG	TG	TRUE	TRUE	GCCAATCATCAGTCCTCTTTCTA	CACACCCTGAGATTCATTGATTT
chr16_29714174_29714174_T	T	TRUE	FALSE	CAGAAAATTATCAAGGGCAAAAA	GGCAAGAGAATCACTTAAGGACA
chr17_19881411_19881411_C	C	TRUE	FALSE	AGACCCAGTCTCTGCAAAACTAA	GGGTTCTCCTGAGGAAGCAG
chr17_76662601_76662601_A	A	TRUE	TRUE	GATCACAGTATTTCTGCCTCAGC	CATCTAGAGGATCAAGCTCAACC
chr19_13263020_13263020_T	T	TRUE	FALSE	GTAGGGCGGAGATTTTCTATCTG	GTGATTCGTACCAAGCCATTC
chr1_9952871_9952871_A	A	TRUE	FALSE	AACAGGCTGTCAGCATAAAGGTA	GTTTCCTCCTTCCTTGGATTATG
chr2_136873828_136873828_A	A	TRUE	FALSE	GAGGGTTTCAAAGTCACATCTTG	AAGTTTTTGCGCTTTAGGAGAAT
chr21_46333509_46333509_C	C	TRUE	FALSE	CTTGATTCCAGCAGGATTGG	AGTGCACTGCCTTACCTCCT
chr2_165928668_165928668_A	A	TRUE	TRUE	TCGATAAGTCAAAGAAGAAGTTGC	CCAGTTCATTAAATTTTGCTCGT
chr22_39416982_39416982_G	G	TRUE	TRUE	CTGTCTGTCTTGATGGTGGAGT	GGACGCTTTATCTTCCTCAGTCT
chr2_54724504_54724504_T	T	TRUE	TRUE	CTGTCTTGGGTTCTCTGAAAATG	ACTCCCTGGTGGTTAGAGTGAGT
chr2_855505_855505_A	A	TRUE	TRUE	ATGGGAGAGTGTGTAGCTCTCAA	CAGTGAAATCACAGCCAGTGTAG
chr2_85926078_85926078_GCG	GCG	TRUE	TRUE	CAATAAATGTCTGTCGTGTGTCC	TTTCTGTTCCTTTTCACCAAAAA
chr5_179051010_179051010_C	C	TRUE	FALSE	GATTATTACACGCTTGGGTTTTG	CAGAGCTCGGGACTCCTATACA
chr6_5984400_5984400_TT	TT	TRUE	TRUE	CCACTCAAGACCTTTAGGAGACA	CGGAGATACCTTCTCTGACCTTT
chr7_148149548_148149548_CT	CT	FALSE	TRUE	TGCAGTCTACACAGCTCTTCAAA	AGGGAATTGTCCTACAGGATCTC
chr8_104367284_104367284_A	A	TRUE	TRUE	CTGAGATCATGCTCCTGTACTCC	TATGTCTCCAGGAATCTCTGCAT
chr9_95121195_95121195_C	C	FALSE	FALSE	TTTCAGGGATGCTGTCTATTCAT	GTGGCACATGCCTTTAATCCTA

Supplementary Table 3:

Insertions tested with Sanger sequencing of targeted PCR products

	id	Ref Reads	Ins Reads	PCR Primer 1f sequence	PCR Primer 2r sequence	Primer 1f Result	Primer 2r Result	Pipeline-predicted Insertion	Sanger-detected Insertion	Matches Prediction
	chr20_10427169_10427169_TGTAGGTAAGTGCA	2	22	GAAACCCCGTCTCTATTAAAAACA	TCACCAAGAAGGATATACAGATGG	Heterozygous insertion	Heterozygous insertion	TGTAGGTAAGTGCA	TGTAGGTAAGTGCA	TRUE
	chr3_133684644_133684644_GGGAAAGA	13	10	ATTAGACCCACGGCTATAAGGAG	AAGCTCAAGAATGGGGACTGTA	Heterozygous insertion	Heterozygous insertion	GGGAAAGA	GGGAAAGA	TRUE
	chr4_74473437_74473437_TCTTTCTTTTAAGAT	13	14	ACCCACTGTCCTGAACTTTGATA	CTGTCCTACTCTTGTGGAGCTTG	Homozygous insertion	Homozygous insertion	TCTTTCTTTTAAGAT	Deletion of AGAACCCTCCA; insertion of GAT	FALSE
	chr7_38272793_38272793_ACTG	12	3	AGGGTGGAGAGTGTGTTTGATAG	TGATTGAATCTCTTTAGGGCAAA	Heterozygous insertion	Heterozygous insertion	ACTG	TCCACCACCACTG	FALSE
	chr8_117914942_117914942_AGCAGAGCATA	3	12	TTCTTAAAGTGCTTCATGGCTTATT	AAATTTTGACCACTTTTCCAAGC	Sequencing failed	Homozygous insertion	AGCAGAGCATA	AGAGCATAAGC	FALSE
	chr9_93940142_93940142_GATCTTACAAAAGTGCTT	18	32	CAATGTGTTTTCTGAGGGAAAAA	GTTCTCTGGGTTTTTGATCGTC	Sequencing failed	Heterozygous insertion	GATCTTACAAAAGTGCTT	GATCTTACAAAAGTGCTT	TRUE
**Jurkat**	chr1_112013952_112013952_T	16	13	TAATCTCTCAGCCGGTCTATTCA	TGTGATTGTTAAGTGTGCTGTGA	Heterozygous insertion	Heterozygous insertion	TT	T	FALSE
	chr1_151797452_151797452_T	15	4	CTTAGGGCACTGGGCTCTCT	GGTCAGTCACCCATTTGACC	No Insertion	No Insertion	T	N/A	N/A
	chr10_102414193_102414193_CTT	6	26	CGTCTCTGTCTCTCTGTGTGACTC	TGAGAGCCTCGGCTTAATCTTAT	Homozygous insertion	Homozygous insertion	CTT	CTT	TRUE
	chr12_49450338_49450338_T	48	3	CAGGGTTTCCACACACATACAT	CTGTCTCATCTGTCCCAGTCATT	No Insertion	No Insertion	T	N/A	N/A
	chr12_49452613_49452613_C	12	6	ATACACACCCTTTCTCCCCAATA	AAGGGAGAGTGTGTGGAAACTG	No Insertion	No Insertion	C	N/A	N/A
	chr2_61765428_61765428_C	67	10	AAGGCTCGCCTAAACTTTCC	AATCGCTGCTGAAAAGAGGTAG	No Insertion	No Insertion	C	N/A	N/A
	chr2_191887806_191887806_C	64	16	TTTCAGGAACACTTGGTTCATCT	CACGCTTCCTCCATAGCATC	No Insertion	No Insertion	C	N/A	N/A
	chr6_14771118_14771118_AA	2	4	GGAGAGGTGTTCTGTCTGCTTC	CTTCATAATGCTTTTGACCTTGG	Heterozygous insertion	Heterozygous insertion	AA	AA	TRUE
	chr12_133235615_133235615_A	0	8	CCCTCCTCACACAGAAGTAAAGA	CTGAGTTGCCGCTACATCATCT	Sequencing failed	Heterozygous insertion	A	A	TRUE

	id	Ins Reads	Ref Reads	PCR Primer 1f sequence	PCR Primer 2r sequence	Primer 1f Result	Primer 2r Result	Pipeline-predicted Insertion	Sanger-detected Insertion	Matches Prediction
	chr10_90039346_90039346_TGAATCTC	12	4	CCAGCATTTATGGAGTGTTTTCT	GGTGGTTTGCTCTAATGTAGTCC	Heterozygous insertion	Heterozygous insertion	TGAATCTC	Deletion of TA; Insertion of ACACTAAGGAAATGAATC	FALSE
	chr2_46658697_46658697_GAATCTTTAACAGTACTGTAAC	11	12	AGGAGAGGCATGACATGATCTAA	AGGCAGGCAGTGGAAAATATAA	Homozygous insertion	Homozygous insertion	GAATCTTTAACAGTACTGTAAC	Deletionof CAGGCATGG; Insertion of TGTAAC	FALSE
	chr4_178362311_178362311_CACTTCAAGCAT	5	16	AGCAAACAATTACAACGTGGACT	TCCATCTGAGGTTGCTAACGTAT	Homozygous insertion	Homozygous insertion	CACTTCAAGCAT	CACTTCAAGCAT	TRUE
	chr6_160437344_160437344_AGTGT	5	13	TAATACAGACTGCTTGGGACTGG	GAAGTGGTACTTGCAGGAAAGAG	Heterozygous insertion	Heterozygous insertion	AGTGT	GTATA	FALSE
**MOLT4**	chr1_32716532_32716532_T	115	29	TGTTTTCCAGCATTCTGTCTGTA	AAGCTTCCTGCCACCATAGAC	No Insertion	No Insertion	T	N/A	N/A
	chr1_32740133_32740133_G	106	26	AAGGCCCCATTATATCTGATGTT	GAGCCTTCGTAGGTAACCAGTG	No Insertion	No Insertion	G	N/A	N/A
	chr1_108504945_108504945_TAGT	4	8	TCTCAGGTTAGGAGATCATGTGC	CTAGGCCGATGAGTTTGTATCAT	Heterozygous insertion	Heterozygous insertion	TAGT	TTAG	FALSE
	chr11_33956762_33956762_CGGTTTAA	0	19	ATTTGCCTAACTCATTGCTTGAA	AGAAACTTCCAAGACCTCCATGT	Heterozygous insertion	Heterozygous insertion	A	Aneuploid	FALSE
	chr11_128481712_128481712_C	53	18	AATGACAGTGCTGGAACTGAGAC	CTTGCACTCTTAGCTGCTCTCAT	No Insertion	No Insertion	C	N/A	N/A
	chr11_33956762_33956762_CGGTTTAA	0	19	GGAGACTAAAGTGTCTGGGACAA	CAGTCTAAGTTGCCTCCATTTCA	Heterozygous insertion	Heterozygous insertion	CGGTTTAA	GTTTAACG	FALSE

	id	Ins Reads	Ref Reads	PCR Primer 1f sequence	PCR Primer 2r sequence	Primer 1f Result	Primer 2r Result	Pipeline-predicted Insertion	Sanger-detected Insertion	Matches Prediction
**Kelly**	chr15_78910599_78910599_T	1		6GGTGTGTGGTTTGCTTGAGAT	CAGATTGGGGTCCACTGACT	Homozygous insertion	Homozygous insertion	T	T	TRUE
	chr1_85085601_85085601_AC	2		11GAGGACAGCCCCAAGAGTAGA	GAGCCTTACTCCTCTAATGCAAA	Homozygous insertion	Homozygous insertion	AC	AC	TRUE

	id	Ins Reads	Ref Reads	PCR Primer 1f sequence	PCR Primer 2r sequence	Primer 1f Result	Primer 2r Result	Pipeline-predicted Insertion	Sanger-detected Insertion	Matches Prediction
	chr11_30881964_30881964_TTGTTT	2		7CCCAGCTCGTTATCCCTTTAC	CAGCTTTGGTGCCATTTACTT	Homozygous insertion	Homozygous insertion	TTGTTT	TTGTTT	TRUE
	chr12_20662752_20662752_TA	2		5GCCCTATAAAAATCCCAGCAG	AATGTATGTCAAGCACATTTGAA	Heterozygous insertion	Heterozygous insertion	TA	TA	TRUE
	chr14_91142151_91142151_T	1		4TCACCAAACCATGGACTAAAAA	CGTGGATTTCAGATCTTGTGC	Homozygous insertion	Homozygous insertion	T	T	TRUE
**SHSY5Y**	chr20_21125275_21125275_ATATTA	0		4AAAGCTCTTGGGGCTAAAATG	AGTCATGGATTGTGGTCCATC	Homozygous insertion	Homozygous insertion	ATATTA	ATATTA	TRUE
	chr2_239008737_239008737_GAGGCGG	0		9TGTGTCCCCAGAGATTCCTA	GGCTCTGGTTACCCGATAAG	Homozygous insertion	Homozygous insertion	GAGGCGG	AGGCGGG	FALSE
	chr2_24306108_24306108_T	0		4GTGTCCCCGATCTTCCAGT	GAACCATCGTCAAGGTTCCTA	Heterozygous insertion	Heterozygous insertion	T	T	TRUE
	chr6_28575297_28575297_TT	1		4CCTTTCTCCCTCCCGTTAAAT	ACCCCATGCTATGACAGACAG	Heterozygous insertion	Heterozygous insertion	TT	T	FALSE
	chr7_35840898_35840898_T	1		20GAGTCGAGCGAGAGCCTGT	GGCTCTCCTCACGTTCAATTC	Sequencing failed	Homozygous insertion	T	C	FALSE

	id	Ins Reads	Ref Reads	PCR Primer 1f sequence	PCR Primer 2r sequence	Primer 1f Result	Primer 2r Result	Pipeline-predicted Insertion	Sanger-detected Insertion	Matches Prediction
**LS174T**	chr12_48337074_48337074_G	4	15	ATCTCCCCTTGCTTACACCAC	TGAACCCCCTGCTTCAGATAC	Heterozygous insertion	Heterozygous insertion	G	G	TRUE
	chr21_40361328_40361328_G	4	9	TTGCCATAAGGAGTCTGTGCT	GCAGGGAAATATGTGAGTGGA	Heterozygous insertion	Heterozygous insertion	G	G	TRUE

Supplementary Table 4:
Insertions tested by comparison with GM12878 Illumina Platinum genome

PREDICTION ID	IPG CHROM	IPG SITE	IPG REFERENCE	IPG REPLACEMENT
chr10_104406046_104406046_TGGGGCC	chr10	104406045	T	TTGGGGCC
chr10_105668044_105668044_G	chr10	105668043	A	AG
chr10_105668180_105668180_TT	chr10	105668179	C	CTT
chr10_105881288_105881288_C	N.A.	N.A.	N.A.	N.A.
chr10_11211532_11211532_G	chr10	11211531	A	AG
chr10_112327679_112327679_A	chr10	112327678	C	CA
chr10_112603247_112603247_A	N.A.	N.A.	N.A.	N.A.
chr10_115616246_115616246_AAAT	chr10	115616245	C	CAAAT
chr10_120924536_120924536_TGTT	chr10	120924535	C	CTGTT
chr10_12171198_12171198_A	chr10	12171197	C	CA
chr10_12306522_12306522_T	chr10	12306521	C	CT
chr10_126407617_126407617_A	chr10	126407616	T	TA
chr10_13390840_13390840_C	chr10	13390839	T	TC
chr10_15002141_15002141_C	chr10	15002140	T	TC
chr10_23055946_23055946_A	chr10	23055945	T	TA
chr10_26728589_26728589_GC	chr10	26728588	A	AGC
chr10_26987106_26987106_TTAA	chr10	26987105	G	GTTAA
chr10_27388796_27388796_G	chr10	27388795	A	AG
chr10_27389841_27389841_A	chr10	27389840	C	CA
chr10_27445440_27445440_T	chr10	27445439	A	AT
chr10_27530606_27530606_G	chr10	27530605	T	TG
chr10_27531665_27531665_CCT	chr10	27531664	A	ACCT
chr10_30990201_30990201_G	chr10	30990200	A	AG
chr10_32622697_32622697_GAG	chr10	32622696	C	CGAG
chr10_38264755_38264755_G	N.A.	N.A.	N.A.	N.A.
chr10_38328116_38328116_T	chr10	38328115	G	GT
chr10_3917656_3917656_A	N.A.	N.A.	N.A.	N.A.
chr10_43916564_43916564_G	chr10	43916563	C	CG
chr10_45460316_45460316_A	chr10	45460315	G	GA
chr10_51372468_51372468_ACTC	N.A.	N.A.	N.A.	N.A.
chr10_51622286_51622286_GAGT	N.A.	N.A.	N.A.	N.A.
chr10_52215492_52215492_C	N.A.	N.A.	N.A.	N.A.
chr10_61639351_61639351_T	chr10	61639350	A	AT
chr10_63703230_63703230_CT	N.A.	N.A.	N.A.	N.A.
chr10_64996753_64996753_A	chr10	64996752	C	CA
chr10_70982137_70982137_CA	chr10	70982136	T	TCA
chr10_72115654_72115654_CGC	chr10	72115653	A	ACGC
chr10_74091593_74091593_T	N.A.	N.A.	N.A.	N.A.
chr10_74091688_74091688_A	chr10	74091687	C	CA
chr10_7534017_7534017_A	chr10	7534016	G	GA
chr10_7534274_7534274_TTCT	chr10	7534273	C	CTTCT
chr10_7534670_7534670_G	chr10	7534669	A	AG
chr10_7534671_7534671_C	N.A.	N.A.	N.A.	N.A.

chr10_75936126_75936126_G	N.A.	N.A.	N.A.	N.A.
chr10_81948162_81948162_CCTTTCT	chr10	81948161	G	GCCTTTCT
chr10_88150988_88150988_T	N.A.	N.A.	N.A.	N.A.
chr10_89625916_89625916_T	N.A.	N.A.	N.A.	N.A.
chr10_89690953_89690953_TTATC	chr10	89690952	T	TTTATC
chr10_89826666_89826666_T	chr10	89826665	A	AT
chr10_89874150_89874150_A	chr10	89874149	G	GA
chr10_92758145_92758145_G	N.A.	N.A.	N.A.	N.A.
chr10_92964092_92964092_A	chr10	92964091	T	TA
chr10_98048361_98048361_A	chr10	98048360	C	CA
chr10_99894577_99894577_T	N.A.	N.A.	N.A.	N.A.
chr1_101704085_101704085_T	chr1	101704084	C	CT
chr1_101823254_101823254_T	chr1	101823253	C	CT
chr1_101854449_101854449_C	chr1	101854448	G	GC
chr1_101872733_101872733_T	chr1	101872732	C	CT
chr11_102187635_102187635_A	chr11	102187634	T	TA
chr11_102217810_102217810_CT	N.A.	N.A.	N.A.	N.A.
chr11_102217811_102217811_TG	N.A.	N.A.	N.A.	N.A.
chr11_102218194_102218194_C	chr11	102218193	G	GC
chr11_108583971_108583971_A	N.A.	N.A.	N.A.	N.A.
chr11_108725456_108725456_T	chr11	108725455	A	AT
chr11_108725702_108725702_A	chr11	108725701	G	GA
chr11_111244484_111244484_T	chr11	111244483	C	CT
chr11_111249414_111249414_C	chr11	111249413	A	AC
chr11_111700256_111700256_T	chr11	111700255	A	AT
chr1_111177850_111177850_TG	N.A.	N.A.	N.A.	N.A.
chr1_111421610_111421610_A	chr1	111421609	G	GA
chr11_117016168_117016168_T	N.A.	N.A.	N.A.	N.A.
chr1_111759431_111759431_ACAC	N.A.	N.A.	N.A.	N.A.
chr11_118868843_118868843_G	chr11	118868842	A	AG
chr11_121107849_121107849_G	N.A.	N.A.	N.A.	N.A.
chr11_121333970_121333970_T	chr11	121333969	A	AT
chr1_112137101_112137101_GGAA	chr1	112137100	C	CGGAA
chr11_123277977_123277977_T	chr11	123277976	A	AT
chr11_124669520_124669520_C	chr11	124669519	T	TC
chr11_128392904_128392904_C	chr11	128392903	T	TC
chr11_130637391_130637391_C	chr11	130637390	A	AC
chr1_113616383_113616383_A	chr1	113616382	G	GA
chr11_14292031_14292031_A	chr11	14292030	C	CA
chr11_14336020_14336020_A	N.A.	N.A.	N.A.	N.A.
chr11_14349361_14349361_A	chr11	14349360	G	GA
chr1_115054290_115054290_T	N.A.	N.A.	N.A.	N.A.
chr1_116917815_116917815_C	N.A.	N.A.	N.A.	N.A.
chr1_117605131_117605131_T	N.A.	N.A.	N.A.	N.A.
chr1_11865285_11865285_C	chr1	11865284	A	AC
chr1_12104257_12104257_A	N.A.	N.A.	N.A.	N.A.
chr1_12217329_12217329_T	chr1	12217328	A	AT
chr11_268181_268181_CT	chr11	268180	C	CCT

chr11_33278347_33278347_GA	chr11	33278346	T	TGA
chr11_3399608_3399608_A	N.A.	N.A.	N.A.	N.A.
chr11_36764355_36764355_CAGTATG	chr11	36764354	C	CCAGTATG
chr11_36765929_36765929_C	chr11	36765928	A	AC
chr11_44588345_44588345_T	chr11	44588344	C	CT
chr1_144908641_144908641_G	N.A.	N.A.	N.A.	N.A.
chr11_47586722_47586722_G	chr11	47586721	T	TG
chr1_150135595_150135595_AC	chr1	150135594	A	AAC
chr1_150536181_150536181_AAG	chr1	150536180	C	CAAG
chr1_150550020_150550020_A	chr1	150550019	T	TA
chr1_153963996_153963996_AG	chr1	153963995	A	AAG
chr1_153964397_153964397_AA	chr1	153964396	C	CAA
chr1_154192464_154192464_T	chr1	154192463	G	GT
chr1_154582755_154582755_ATCTC	chr1	154582754	T	TATCTC
chr1_154973295_154973295_CCCCAC	chr1	154973294	A	ACCCCAC
chr1_155880761_155880761_A	chr1	155880760	C	CA
chr1_155930274_155930274_G	chr1	155930273	A	AG
chr1_156187519_156187519_A	N.A.	N.A.	N.A.	N.A.
chr11_5646946_5646946_GTC	chr11	5646945	G	GGTC
chr1_156571619_156571619_A	chr1	156571618	T	TA
chr1_156630181_156630181_TG	N.A.	N.A.	N.A.	N.A.
chr1_156631567_156631567_AT	chr1	156631566	C	CAT
chr11_5705651_5705651_C	chr11	5705650	A	AC
chr1_158980927_158980927_T	chr1	158980926	C	CT
chr1_158981902_158981902_T	chr1	158981901	A	AT
chr1_159890964_159890964_CC	chr1	159890963	G	GCC
chr1_159891276_159891276_C	chr1	159891275	A	AC
chr1_160444460_160444460_C	N.A.	N.A.	N.A.	N.A.
chr1_160492419_160492419_T	chr1	160492418	C	CT
chr1_160492424_160492424_G	N.A.	N.A.	N.A.	N.A.
chr1_160655900_160655900_T	N.A.	N.A.	N.A.	N.A.
chr1_160681654_160681654_A	N.A.	N.A.	N.A.	N.A.
chr1_160682116_160682116_A	N.A.	N.A.	N.A.	N.A.
chr1_160766180_160766180_T	chr1	160766179	C	CT
chr11_60930746_60930746_CAGTC	chr11	60930745	G	GCAGTC
chr1_161103606_161103606_AG	chr1	161103605	A	AAG
chr1_161147574_161147574_ACTA	chr1	161147573	C	CACTA
chr11_61891898_61891898_TTGTT	chr11	61891897	C	CTTGTT
chr11_62433782_62433782_A	N.A.	N.A.	N.A.	N.A.
chr11_62446870_62446870_G	chr11	62446869	T	TG
chr11_62607951_62607951_A	N.A.	N.A.	N.A.	N.A.
chr11_63618986_63618986_T	chr11	63618985	C	CT
chr11_64619861_64619861_C	chr11	64619860	G	GC
chr11_64647443_64647443_TC	chr11	64647442	G	GTC
chr11_64684306_64684306_G	chr11	64684305	T	TG
chr11_65728675_65728675_A	N.A.	N.A.	N.A.	N.A.
chr11_65728677_65728677_AAAAAC	chr11	65728676	A	AAAAAC,AAAAAAC
chr11_65728677_65728677_AAAAC	N.A.	N.A.	N.A.	N.A.

chr11_65881578_65881578_T	chr11	65881577	G	GT
chr11_67418407_67418407_T	chr11	67418406	G	GT
chr11_67807144_67807144_C	chr11	67807143	A	AC
chr11_67889385_67889385_A	N.A.	N.A.	N.A.	N.A.
chr11_67889386_67889386_C	chr11	67889385	T	TC
chr11_68228379_68228379_G	chr11	68228378	T	TG
chr1_168435506_168435506_T	chr1	168435505	A	AT
chr11_68924077_68924077_TGTT	N.A.	N.A.	N.A.	N.A.
chr11_696768_696768_A	chr11	696767	C	CA
chr1_16970871_16970871_C	N.A.	N.A.	N.A.	N.A.
chr1_172328041_172328041_A	chr1	172328040	G	GA
chr1_172328768_172328768_A	chr1	172328767	T	TA
chr1_172360854_172360854_T	N.A.	N.A.	N.A.	N.A.
chr11_73098833_73098833_T	chr11	73098832	C	CT
chr1_173386661_173386661_G	chr1	173386660	T	TG
chr11_73694543_73694543_G	chr11	73694542	A	AG
chr1_174959537_174959537_A	N.A.	N.A.	N.A.	N.A.
chr1_174968531_174968531_C	chr1	174968530	T	TC
chr1_175111607_175111607_AAAT	chr1	175111606	C	CAAAT
chr11_7553457_7553457_T	chr11	7553456	C	CT
chr11_75535439_75535439_T	N.A.	N.A.	N.A.	N.A.
chr11_75583417_75583417_A	chr11	75583416	C	CA
chr11_77156019_77156019_A	chr11	77156018	C	CA
chr11_77160541_77160541_A	chr11	77160540	C	CA
chr11_77183901_77183901_A	chr11	77183900	C	CA
chr1_179052481_179052481_CT	chr1	179052480	A	ACT
chr1_179052534_179052534_T	chr1	179052533	C	CT
chr1_181003341_181003341_G	chr1	181003340	A	AG
chr11_83323881_83323881_C	chr11	83323880	T	TC
chr11_83325061_83325061_T	chr11	83325060	A	AT
chr1_183812956_183812956_T	chr1	183812955	G	GT
chr1_183867491_183867491_ACCC	chr1	183867490	A	AACCC
chr11_85778821_85778821_AA	chr11	85778820	T	TAA
chr11_8703974_8703974_C	chr11	8703973	G	GC
chr11_88070211_88070211_A	chr11	88070210	G	GA
chr11_88089717_88089717_TTA	chr11	88089716	C	CTTA
chr11_8986841_8986841_AG	chr11	8986840	C	CAG
chr11_94706456_94706456_GT	N.A.	N.A.	N.A.	N.A.
chr11_95846769_95846769_AA	N.A.	N.A.	N.A.	N.A.
chr11_95847189_95847189_A	chr11	95847188	G	GA
chr1_19717566_19717566_T	chr1	19717565	C	CT
chr1_19780412_19780412_GAA	chr1	19780411	T	TGAA
chr1_200596711_200596711_A	chr1	200596710	T	TA
chr1_200989162_200989162_C	N.A.	N.A.	N.A.	N.A.
chr1_201952441_201952441_CCT	N.A.	N.A.	N.A.	N.A.
chr1_203258224_203258224_A	chr1	203258223	T	TA
chr1_205600542_205600542_AAGT	chr1	205600541	C	CAAGT
chr1_206289129_206289129_AA	chr1	206289128	T	TAA

chr1_206670259_206670259_GGAA	chr1	206670258	G	GGGAA
chr1_206807940_206807940_T	N.A.	N.A.	N.A.	N.A.
chr1_206912773_206912773_TCC	chr1	206912772	G	GTCC
chr1_207094367_207094367_T	chr1	207094366	A	AT
chr1_209161696_209161696_C	chr1	209161695	G	GC
chr1_209942591_209942591_G	chr1	209942590	C	CG
chr12_10517739_10517739_A	chr12	10517738	C	CA
chr12_10693164_10693164_C	chr12	10693163	A	AC
chr12_108956527_108956527_G	chr12	108956526	A	AG
chr12_110980618_110980618_A	N.A.	N.A.	N.A.	N.A.
chr12_111015524_111015524_TAAC	chr12	111015523	T	TTAAC
chr12_111016954_111016954_A	N.A.	N.A.	N.A.	N.A.
chr12_111017836_111017836_A	N.A.	N.A.	N.A.	N.A.
chr12_11912718_11912718_C	chr12	11912717	A	AC
chr12_120966609_120966609_A	N.A.	N.A.	N.A.	N.A.
chr12_120966609_120966609_AA	chr12	120966608	C	CAA
chr12_121341076_121341076_C	chr12	121341075	T	TC
chr12_12135447_12135447_TA	chr12	12135446	C	CTA
chr12_121567332_121567332_TTT	N.A.	N.A.	N.A.	N.A.
chr1_212209368_212209368_C	chr1	212209367	T	TC
chr12_122102633_122102633_T	N.A.	N.A.	N.A.	N.A.
chr12_122898197_122898197_A	N.A.	N.A.	N.A.	N.A.
chr12_123210262_123210262_A	chr12	123210261	C	CA
chr12_125034711_125034711_G	chr12	125034710	A	AG
chr12_125347765_125347765_CA	chr12	125347764	T	TCA
chr12_14548253_14548253_G	chr12	14548252	T	TG
chr12_22696643_22696643_TG	chr12	22696642	T	TTG
chr1_223913812_223913812_TT	chr1	223913811	C	CTT
chr1_224302155_224302155_GAG	chr1	224302154	T	TGAG
chr1_224372729_224372729_AGAT	chr1	224372728	C	CAGAT
chr12_25212204_25212204_T	chr12	25212203	A	AT
chr12_25253760_25253760_T	chr12	25253759	C	CT
chr12_25348488_25348488_GGGA	chr12	25348487	T	TGGGA
chr1_225629764_225629764_T	N.A.	N.A.	N.A.	N.A.
chr1_226591875_226591875_CT	N.A.	N.A.	N.A.	N.A.
chr1_226591876_226591876_TC	chr1	226591875	A	ATC
chr1_226595083_226595083_G	chr1	226595082	A	AG
chr1_226595404_226595404_GGGCCC	chr1	226595403	A	AGGGCCC
chr1_227183204_227183204_CA	chr1	227183203	C	CCA
chr12_27332525_27332525_TT	chr12	27332524	A	ATT
chr12_28014791_28014791_A	chr12	28014790	C	CA
chr12_31883015_31883015_GGTTGG	chr12	31883014	T	TGGTTGG
chr12_32081480_32081480_TTTTT	N.A.	N.A.	N.A.	N.A.
chr1_23359000_23359000_G	chr1	23358999	T	TG
chr1_234635299_234635299_T	chr1	234635298	G	GT
chr1_234735007_234735007_C	chr1	234735006	G	GC
chr1_234739685_234739685_TCTC	chr1	234739684	A	ATCTC
chr1_234867199_234867199_A	chr1	234867198	G	GA

chr1_235159624_235159624_G	chr1	235159623	A	AG
chr1_236324218_236324218_T	chr1	236324217	G	GT
chr1_23884207_23884207_G	chr1	23884206	A	AG
chr1_23886339_23886339_C	chr1	23886338	G	GC
chr12_39168415_39168415_T	chr12	39168414	A	AT
chr1_24049482_24049482_TA	chr1	24049481	T	TTA
chr1_241715313_241715313_GG	chr1	241715312	A	AGG
chr12_4227474_4227474_GT	N.A.	N.A.	N.A.	N.A.
chr1_24231681_24231681_T	N.A.	N.A.	N.A.	N.A.
chr1_24232610_24232610_G	chr1	24232609	A	AG
chr12_42492259_42492259_CA	chr12	42492258	T	TCA
chr1_24306357_24306357_T	N.A.	N.A.	N.A.	N.A.
chr1_24306358_24306358_G	chr1	24306357	T	TG
chr12_4429705_4429705_GGGGGA	chr12	4429704	T	TGGGGGA
chr12_4429711_4429711_GGGGGC	N.A.	N.A.	N.A.	N.A.
chr1_244462517_244462517_AG	chr1	244462516	C	CAG
chr1_245083540_245083540_T	N.A.	N.A.	N.A.	N.A.
chr1_246792702_246792702_TGTC	chr1	246792701	A	ATGTC
chr1_24719349_24719349_A	N.A.	N.A.	N.A.	N.A.
chr1_247290662_247290662_GGTC	N.A.	N.A.	N.A.	N.A.
chr12_48100244_48100244_T	N.A.	N.A.	N.A.	N.A.
chr1_248599197_248599197_AC	N.A.	N.A.	N.A.	N.A.
chr1_248611450_248611450_A	chr1	248611449	G	GA
chr1_248611504_248611504_A	N.A.	N.A.	N.A.	N.A.
chr1_248611570_248611570_GAA	chr1	248611569	T	TGAA
chr1_248611599_248611599_CA	N.A.	N.A.	N.A.	N.A.
chr1_248807156_248807156_T	chr1	248807155	C	CT
chr1_248819475_248819475_TT	N.A.	N.A.	N.A.	N.A.
chr12_49246308_49246308_AAC	chr12	49246307	T	TAAC
chr1_24968963_24968963_AAC	chr1	24968962	A	AAAC
chr12_50016837_50016837_T	N.A.	N.A.	N.A.	N.A.
chr12_50794257_50794257_T	chr12	50794256	G	GT
chr12_50927398_50927398_T	chr12	50927397	C	CT
chr1_25250920_25250920_T	chr1	25250919	G	GT
chr12_53835781_53835781_G	N.A.	N.A.	N.A.	N.A.
chr12_56324560_56324560_ACA	chr12	56324559	T	TACA
chr12_57079895_57079895_TCCTA	chr12	57079894	T	TTCCTA
chr1_25773709_25773709_A	chr1	25773708	T	TA
chr12_57873970_57873970_T	chr12	57873969	C	CT
chr12_58139254_58139254_A	chr12	58139253	C	CA
chr12_59990297_59990297_G	chr12	59990296	A	AG
chr12_64798671_64798671_G	chr12	64798670	C	CG
chr12_6643506_6643506_A	N.A.	N.A.	N.A.	N.A.
chr12_6644068_6644068_C	N.A.	N.A.	N.A.	N.A.
chr12_66443939_66443939_T	chr12	66443938	C	CT
chr12_76479911_76479911_T	chr12	76479910	C	CT
chr12_76742474_76742474_C	N.A.	N.A.	N.A.	N.A.
chr12_77157590_77157590_C	chr12	77157589	G	GC

chr1_27756286_27756286_A	N.A.	N.A.	N.A.	N.A.
chr12_785783_785783_AAG	chr12	785782	C	CAAG
chr1_27985078_27985078_T	chr1	27985077	A	AT
chr1_27989761_27989761_GA	chr1	27989760	T	TGA
chr12_81171705_81171705_A	N.A.	N.A.	N.A.	N.A.
chr12_81173237_81173237_C	chr12	81173236	T	TC
chr12_831816_831816_G	chr12	831815	T	TG
chr1_28573708_28573708_G	chr1	28573707	A	AG
chr12_90353392_90353392_AA	N.A.	N.A.	N.A.	N.A.
chr12_92837318_92837318_A	chr12	92837317	T	TA
chr12_92886025_92886025_A	chr12	92886024	C	CA
chr12_92899521_92899521_GC	chr12	92899520	T	TGC
chr12_92937365_92937365_TT	chr12	92937364	G	GTT
chr12_93690333_93690333_AATGACTG	chr12	93690332	A	AAATGACTG
chr12_94676937_94676937_G	chr12	94676936	A	AG
chr12_96253004_96253004_G	chr12	96253003	A	AG
chr12_9881545_9881545_C	chr12	9881544	A	AC
chr12_98898105_98898105_T	chr12	98898104	C	CT
chr13_103683747_103683747_C	chr13	103683746	A	AC
chr13_103683951_103683951_A	chr13	103683950	G	GA
chr13_105636073_105636073_T	N.A.	N.A.	N.A.	N.A.
chr13_107219671_107219671_TTAT	chr13	107219670	C	CTTAT
chr13_20141054_20141054_A	chr13	20141053	G	GA
chr13_24152517_24152517_ATG	chr13	24152516	A	AATG
chr13_24154521_24154521_G	chr13	24154520	C	CG
chr13_25199984_25199984_A	N.A.	N.A.	N.A.	N.A.
chr13_26760387_26760387_A	N.A.	N.A.	N.A.	N.A.
chr13_27722265_27722265_A	chr13	27722264	C	CA
chr13_33725273_33725273_A	chr13	33725272	G	GA
chr13_33757849_33757849_G	chr13	33757848	T	TG
chr13_34304115_34304115_G	chr13	34304114	A	AG
chr13_41560790_41560790_TCTG	chr13	41560789	C	CTCTG
chr13_41579615_41579615_A	N.A.	N.A.	N.A.	N.A.
chr13_41580461_41580461_T	chr13	41580460	G	GT
chr13_42039690_42039690_CC	N.A.	N.A.	N.A.	N.A.
chr13_42039692_42039692_GT	N.A.	N.A.	N.A.	N.A.
chr13_42076797_42076797_G	N.A.	N.A.	N.A.	N.A.
chr13_42988314_42988314_G	N.A.	N.A.	N.A.	N.A.
chr13_44832846_44832846_A	chr13	44832845	T	TA
chr13_46752042_46752042_A	N.A.	N.A.	N.A.	N.A.
chr13_46964187_46964187_G	chr13	46964186	C	CG
chr13_50571929_50571929_T	chr13	50571928	C	CT
chr13_75940589_75940589_A	chr13	75940588	G	GA
chr13_78304037_78304037_TG	chr13	78304036	C	CTG
chr13_79961428_79961428_A	chr13	79961427	T	TA
chr13_79976161_79976161_A	chr13	79976160	G	GA
chr13_95875673_95875673_CT	chr13	95875672	A	ACT
chr13_95878393_95878393_GC	N.A.	N.A.	N.A.	N.A.

chr13_95913887_95913887_A	N.A.	N.A.	N.A.	N.A.
chr13_95934060_95934060_A	N.A.	N.A.	N.A.	N.A.
chr13_99164974_99164974_A	chr13	99164973	C	CA
chr13_99164977_99164977_G	N.A.	N.A.	N.A.	N.A.
chr13_99167558_99167558_CGC	N.A.	N.A.	N.A.	N.A.
chr14_102414744_102414744_AG	chr14	102414743	C	CAG
chr14_105260907_105260907_G	chr14	105260906	T	TG
chr14_106036870_106036870_AAAC	chr14	106036869	T	TAAAC
chr14_106041089_106041089_T	N.A.	N.A.	N.A.	N.A.
chr14_106043568_106043568_GC	N.A.	N.A.	N.A.	N.A.
chr14_106163388_106163388_C	N.A.	N.A.	N.A.	N.A.
chr14_106163391_106163391_A	N.A.	N.A.	N.A.	N.A.
chr14_106329737_106329737_G	N.A.	N.A.	N.A.	N.A.
chr14_106329739_106329739_G	N.A.	N.A.	N.A.	N.A.
chr14_107176288_107176288_C	chr14	107176287	G	GC
chr1_41159723_41159723_G	chr1	41159722	T	TG
chr1_42089576_42089576_A	chr1	42089575	G	GA
chr14_20959290_20959290_A	chr14	20959289	T	TA
chr14_22946409_22946409_G	chr14	22946408	T	TG
chr14_22954206_22954206_G	chr14	22954205	T	TG
chr14_22960669_22960669_T	chr14	22960668	C	CT
chr14_22963633_22963633_C	chr14	22963632	G	GC
chr14_22964644_22964644_G	chr14	22964643	T	TG
chr14_22964841_22964841_G	chr14	22964840	T	TG
chr14_22974863_22974863_T	chr14	22974862	A	AT
chr14_22975345_22975345_C	chr14	22975344	G	GC
chr14_22977873_22977873_G	chr14	22977872	A	AG
chr14_22978056_22978056_C	chr14	22978055	G	GC
chr14_22978452_22978452_C	chr14	22978451	G	GC
chr14_22980209_22980209_G	N.A.	N.A.	N.A.	N.A.
chr14_22982070_22982070_G	chr14	22982069	A	AG
chr14_22984432_22984432_G	chr14	22984431	T	TG
chr14_22989653_22989653_A	chr14	22989652	G	GA
chr14_22990931_22990931_T	chr14	22990930	A	AT
chr14_22992299_22992299_A	chr14	22992298	C	CA
chr14_22993120_22993120_C	chr14	22993119	T	TC
chr14_22993279_22993279_G	chr14	22993278	A	AG
chr14_22995509_22995509_G	chr14	22995508	T	TG
chr14_23001923_23001923_A	chr14	23001922	G	GA
chr14_23006116_23006116_CA	chr14	23006115	G	GCA
chr14_23007069_23007069_G	chr14	23007068	A	AG
chr14_23018265_23018265_C	chr14	23018264	T	TC
chr14_23018291_23018291_A	chr14	23018290	C	CA
chr14_23018402_23018402_G	chr14	23018401	C	CG
chr14_23020689_23020689_C	chr14	23020688	G	GC
chr14_23023763_23023763_G	chr14	23023762	T	TG
chr14_23023859_23023859_G	chr14	23023858	T	TG
chr14_23024029_23024029_GC	N.A.	N.A.	N.A.	N.A.

chr14_23024074_23024074_C	chr14	23024073	T	TC
chr14_23026207_23026207_C	chr14	23026206	T	TC
chr14_23026214_23026214_C	N.A.	N.A.	N.A.	N.A.
chr14_23026370_23026370_G	chr14	23026369	C	CG
chr14_23034347_23034347_CT	chr14	23034346	A	ACT
chr14_23268546_23268546_GTGG	chr14	23268545	T	TGTGG
chr14_23274373_23274373_A	chr14	23274372	C	CA
chr14_23299010_23299010_C	chr14	23299009	G	GC
chr14_23357764_23357764_A	N.A.	N.A.	N.A.	N.A.
chr1_43312494_43312494_G	chr1	43312493	C	CG
chr14_35342801_35342801_TCTGAT	N.A.	N.A.	N.A.	N.A.
chr14_35342984_35342984_GA	chr14	35342983	C	CGA
chr14_35343465_35343465_C	chr14	35343464	A	AC
chr14_35873370_35873370_G	chr14	35873369	T	TG
chr14_39579596_39579596_AGTA	chr14	39579595	T	TAGTA
chr1_44250786_44250786_A	chr1	44250785	T	TA
chr14_50550134_50550134_TCTG	chr14	50550133	C	CTCTG
chr14_50697731_50697731_A	N.A.	N.A.	N.A.	N.A.
chr1_45188747_45188747_A	chr1	45188746	C	CA
chr14_52314282_52314282_A	chr14	52314281	T	TA
chr14_55539162_55539162_T	N.A.	N.A.	N.A.	N.A.
chr14_55571011_55571011_T	chr14	55571010	C	CT
chr1_46011377_46011377_CA	chr1	46011376	G	GCA
chr14_61905899_61905899_T	N.A.	N.A.	N.A.	N.A.
chr14_61907824_61907824_C	chr14	61907823	G	GC
chr1_46216748_46216748_TC	chr1	46216747	G	GTC
chr1_46216749_46216749_CG	N.A.	N.A.	N.A.	N.A.
chr14_62218317_62218317_A	chr14	62218316	T	TA
chr14_64684460_64684460_T	chr14	64684459	G	GT
chr14_65749190_65749190_G	chr14	65749189	T	TG
chr14_68745970_68745970_G	N.A.	N.A.	N.A.	N.A.
chr14_68780648_68780648_A	chr14	68780647	C	CA
chr14_69259534_69259534_G	chr14	69259533	T	TG
chr14_71120448_71120448_C	chr14	71120447	A	AC
chr14_71135871_71135871_A	N.A.	N.A.	N.A.	N.A.
chr14_71939073_71939073_A	chr14	71939072	C	CA
chr14_72021065_72021065_AAAG	chr14	72021064	T	TAAAG
chr14_74036675_74036675_C	chr14	74036674	G	GC
chr14_77499686_77499686_G	N.A.	N.A.	N.A.	N.A.
chr14_77502690_77502690_A	N.A.	N.A.	N.A.	N.A.
chr14_77547553_77547553_C	chr14	77547552	T	TC
chr14_81936118_81936118_TTT	chr14	81936117	A	ATTT
chr14_89775901_89775901_T	chr14	89775900	A	AT
chr14_89798212_89798212_A	chr14	89798211	T	TA
chr14_89798213_89798213_T	N.A.	N.A.	N.A.	N.A.
chr14_89830607_89830607_A	chr14	89830606	G	GA
chr14_89850142_89850142_T	chr14	89850141	A	AT
chr14_89850668_89850668_GAAAC	chr14	89850667	A	AGAAAC

chr14_91884614_91884614_G	chr14	91884613	A	AG
chr14_92589053_92589053_T	chr14	92589052	A	AT
chr14_93029976_93029976_A	chr14	93029975	T	TA
chr14_93261239_93261239_CT	chr14	93261238	A	ACT
chr14_94435216_94435216_TGAA	chr14	94435215	G	GTGAA
chr14_96127708_96127708_CT	chr14	96127707	G	GCT
chr15_101463036_101463036_C	chr15	101463035	T	TC
chr1_51433822_51433822_C	chr1	51433821	G	GC
chr1_51433864_51433864_C	chr1	51433863	G	GC
chr1_51976405_51976405_T	N.A.	N.A.	N.A.	N.A.
chr15_25682998_25682998_G	chr15	25682997	A	AG
chr15_31645630_31645630_G	chr15	31645629	T	TG
chr15_34331613_34331613_A	chr15	34331612	G	GA
chr1_53791169_53791169_G	N.A.	N.A.	N.A.	N.A.
chr15_40390967_40390967_A	N.A.	N.A.	N.A.	N.A.
chr15_41233534_41233534_C	chr15	41233533	T	TC
chr15_42567780_42567780_T	chr15	42567779	A	AT
chr15_44830488_44830488_T	N.A.	N.A.	N.A.	N.A.
chr15_45028920_45028920_T	N.A.	N.A.	N.A.	N.A.
chr15_45030116_45030116_G	N.A.	N.A.	N.A.	N.A.
chr15_49087107_49087107_C	N.A.	N.A.	N.A.	N.A.
chr15_49912832_49912832_A	N.A.	N.A.	N.A.	N.A.
chr15_50717024_50717024_T	chr15	50717023	G	GT
chr15_51176880_51176880_GTACT	chr15	51176879	C	CGTACT
chr15_52028953_52028953_CACCTA	chr15	52028952	T	TCACCTA
chr15_52273669_52273669_A	chr15	52273668	C	CA
chr15_52673383_52673383_T	chr15	52673382	G	GT
chr15_55611065_55611065_A	N.A.	N.A.	N.A.	N.A.
chr15_56662175_56662175_T	N.A.	N.A.	N.A.	N.A.
chr15_58751601_58751601_TTT	chr15	58751600	C	CTTT
chr15_59529835_59529835_T	chr15	59529834	A	AT
chr15_59594973_59594973_A	chr15	59594972	G	GA
chr15_59702745_59702745_A	chr15	59702744	G	GA
chr15_59980714_59980714_AC	chr15	59980713	G	GAC
chr15_60771439_60771439_C	chr15	60771438	G	GC
chr15_60941452_60941452_G	N.A.	N.A.	N.A.	N.A.
chr15_63792759_63792759_T	chr15	63792758	G	GT
chr15_64736207_64736207_TT	chr15	64736206	G	GTT
chr15_64928081_64928081_TA	chr15	64928080	C	CTA
chr15_65588679_65588679_TTCACT	chr15	65588678	A	ATTCACT
chr15_66682529_66682529_G	N.A.	N.A.	N.A.	N.A.
chr15_67374469_67374469_G	chr15	67374468	C	CG
chr15_67814718_67814718_A	N.A.	N.A.	N.A.	N.A.
chr15_68155323_68155323_CA	chr15	68155322	T	TCA
chr15_68230152_68230152_AGA	chr15	68230151	T	TAGA
chr15_68239460_68239460_T	chr15	68239459	G	GT
chr15_69109999_69109999_G	chr15	69109998	A	AG
chr15_69112972_69112972_CA	chr15	69112971	G	GCA

chr15_70784965_70784965_G	chr15	70784964	T	TG
chr15_73031999_73031999_AAG	chr15	73031998	A	AAAG
chr15_73032001_73032001_GAC	N.A.	N.A.	N.A.	N.A.
chr15_74679076_74679076_T	chr15	74679075	C	CT
chr15_75422610_75422610_TT	N.A.	N.A.	N.A.	N.A.
chr15_77712967_77712967_A	N.A.	N.A.	N.A.	N.A.
chr15_77712967_77712967_AA	N.A.	N.A.	N.A.	N.A.
chr15_78357098_78357098_A	chr15	78357097	G	GA
chr15_80259564_80259564_T	N.A.	N.A.	N.A.	N.A.
chr15_81592976_81592976_GT	chr15	81592975	A	AGT
chr15_85277591_85277591_C	chr15	85277590	T	TC
chr15_85944540_85944540_T	chr15	85944539	G	GT
chr15_86245055_86245055_C	chr15	86245054	A	AC
chr15_86255775_86255775_TT	N.A.	N.A.	N.A.	N.A.
chr15_89164230_89164230_T	chr15	89164229	G	GT
chr1_59038460_59038460_AGTA	chr1	59038459	C	CAGTA
chr15_93353744_93353744_AA	N.A.	N.A.	N.A.	N.A.
chr15_99630417_99630417_G	chr15	99630416	T	TG
chr15_99791269_99791269_A	chr15	99791268	T	TA
chr1_60856451_60856451_TT	N.A.	N.A.	N.A.	N.A.
chr16_10480713_10480713_T	chr16	10480712	A	AT
chr16_11043461_11043461_AG	chr16	11043460	C	CAG
chr16_11198133_11198133_T	chr16	11198132	A	AT
chr16_11319328_11319328_CC	chr16	11319327	G	GCC
chr16_11862377_11862377_A	chr16	11862376	G	GA
chr16_11877848_11877848_TGGG	chr16	11877847	C	CTGGG
chr16_15734782_15734782_A	N.A.	N.A.	N.A.	N.A.
chr16_18774415_18774415_TCAATA	chr16	18774414	C	CTCAATA
chr16_19536641_19536641_AG	chr16	19536640	C	CAG
chr16_2059177_2059177_CCCGG	N.A.	N.A.	N.A.	N.A.
chr16_21657480_21657480_T	chr16	21657479	C	CT
chr16_24990137_24990137_A	chr16	24990136	G	GA
chr16_24993417_24993417_T	N.A.	N.A.	N.A.	N.A.
chr16_25029395_25029395_A	chr16	25029394	G	GA
chr16_27439521_27439521_AAG	chr16	27439520	C	CAAG
chr16_2801995_2801995_G	N.A.	N.A.	N.A.	N.A.
chr16_28192840_28192840_T	chr16	28192839	G	GT
chr16_28197994_28197994_T	N.A.	N.A.	N.A.	N.A.
chr16_28200935_28200935_CAAA	chr16	28200934	G	GCAAA
chr16_29673939_29673939_AA	N.A.	N.A.	N.A.	N.A.
chr16_30441724_30441724_TCTC	chr16	30441723	A	ATCTC
chr16_30884413_30884413_A	chr16	30884412	C	CA
chr16_31192240_31192240_T	chr16	31192239	C	CT
chr16_31889954_31889954_A	chr16	31889953	T	TA
chr16_3929032_3929032_TAACA	chr16	3929031	T	TTAACA
chr16_4475594_4475594_C	N.A.	N.A.	N.A.	N.A.
chr16_4664413_4664413_G	chr16	4664412	T	TG
chr16_50310858_50310858_T	N.A.	N.A.	N.A.	N.A.

chr16_5122192_5122192_G	chr16	5122191	C	CG
chr16_53124914_53124914_C	chr16	53124913	A	AC
chr16_57073468_57073468_T	chr16	57073467	C	CT
chr1_66749245_66749245_ACTT	chr1	66749244	A	AACTT
chr16_70557531_70557531_TT	chr16	70557530	G	GTT
chr16_70558406_70558406_T	chr16	70558405	G	GT
chr16_71842108_71842108_A	N.A.	N.A.	N.A.	N.A.
chr16_71879596_71879596_G	chr16	71879595	T	TG
chr16_75467750_75467750_G	chr16	75467749	T	TG
chr16_75605579_75605579_G	N.A.	N.A.	N.A.	N.A.
chr16_81815850_81815850_TG	chr16	81815849	A	ATG
chr16_84627101_84627101_AT	chr16	84627100	C	CAT
chr16_84683103_84683103_TCAG	chr16	84683102	C	CTCAG
chr16_85589768_85589768_T	chr16	85589767	A	AT
chr16_86023061_86023061_GA	chr16	86023060	G	GGA
chr16_87758707_87758707_G	chr16	87758706	T	TG
chr16_87985641_87985641_GC	chr16	87985640	G	GGC
chr16_89552856_89552856_A	N.A.	N.A.	N.A.	N.A.
chr16_9056916_9056916_C	chr16	9056915	G	GC
chr17_10601134_10601134_G	chr17	10601133	C	CG
chr17_1102790_1102790_G	chr17	1102789	T	TG
chr17_1117634_1117634_TA	chr17	1117633	G	GTA
chr17_11924664_11924664_C	N.A.	N.A.	N.A.	N.A.
chr17_12335288_12335288_T	N.A.	N.A.	N.A.	N.A.
chr17_1510428_1510428_CAA	chr17	1510427	G	GCAA
chr17_18762170_18762170_GAG	chr17	18762169	T	TGAG
chr17_20435964_20435964_GA	N.A.	N.A.	N.A.	N.A.
chr17_2118507_2118507_AC	chr17	2118506	A	AAC
chr17_2127981_2127981_A	chr17	2127980	G	GA
chr17_2168671_2168671_A	chr17	2168670	G	GA
chr17_26646435_26646435_C	chr17	26646434	G	GC
chr17_27047244_27047244_G	chr17	27047243	T	TG
chr17_27049151_27049151_G	chr17	27049150	A	AG
chr17_27760725_27760725_G	chr17	27760724	A	AG
chr17_28034171_28034171_A	N.A.	N.A.	N.A.	N.A.
chr17_28255827_28255827_C	N.A.	N.A.	N.A.	N.A.
chr17_29819885_29819885_C	chr17	29819884	A	AC
chr17_29835738_29835738_C	chr17	29835737	G	GC
chr17_30263592_30263592_A	N.A.	N.A.	N.A.	N.A.
chr17_30470029_30470029_C	chr17	30470028	G	GC
chr17_33776088_33776088_AAC	N.A.	N.A.	N.A.	N.A.
chr17_36180236_36180236_TT	chr17	36180235	A	ATT
chr17_37056001_37056001_T	N.A.	N.A.	N.A.	N.A.
chr17_37463550_37463550_A	chr17	37463549	T	TA
chr17_37975215_37975215_TTCTA	chr17	37975214	C	CTTCTA
chr17_38020059_38020059_C	chr17	38020058	A	AC
chr17_38770642_38770642_A	chr17	38770641	T	TA
chr17_41150101_41150101_C	chr17	41150100	T	TC

chr17_42422752_42422752_G	chr17	42422751	C	CG
chr17_43380218_43380218_T	chr17	43380217	C	CT
chr17_43390091_43390091_G	chr17	43390090	A	AG
chr17_43661576_43661576_TA	N.A.	N.A.	N.A.	N.A.
chr17_46189236_46189236_T	N.A.	N.A.	N.A.	N.A.
chr17_46912404_46912404_C	chr17	46912403	G	GC
chr17_47438113_47438113_A	N.A.	N.A.	N.A.	N.A.
chr17_48229075_48229075_GTTTA	chr17	48229074	C	CGTTTA
chr17_48588422_48588422_A	chr17	48588421	G	GA
chr17_49197655_49197655_C	chr17	49197654	A	AC
chr17_54990653_54990653_T	chr17	54990652	C	CT
chr17_57925197_57925197_A	chr17	57925196	G	GA
chr17_61818486_61818486_TTATT	N.A.	N.A.	N.A.	N.A.
chr17_62979858_62979858_A	chr17	62979857	C	CA
chr17_65565230_65565230_GA	chr17	65565229	G	GGA
chr17_65566896_65566896_T	chr17	65566895	A	AT
chr17_7145493_7145493_A	chr17	7145492	C	CA
chr17_73311779_73311779_CAC	chr17	73311778	T	TCAC
chr17_73401042_73401042_T	chr17	73401041	G	GT
chr17_7359357_7359357_T	chr17	7359356	G	GT
chr17_73682029_73682029_T	chr17	73682028	A	AT
chr17_74068230_74068230_C	chr17	74068229	A	AC
chr17_74248918_74248918_T	chr17	74248917	A	AT
chr17_74248920_74248920_G	N.A.	N.A.	N.A.	N.A.
chr17_74488751_74488751_C	N.A.	N.A.	N.A.	N.A.
chr17_74512017_74512017_T	N.A.	N.A.	N.A.	N.A.
chr17_7476919_7476919_C	chr17	7476918	T	TC
chr17_75455628_75455628_A	chr17	75455627	T	TA
chr17_75458222_75458222_AG	chr17	75458221	C	CAG
chr17_76149574_76149574_A	chr17	76149573	G	GA
chr17_76351705_76351705_T	chr17	76351704	G	GT
chr17_76481410_76481410_T	chr17	76481409	A	AT
chr17_76689865_76689865_T	N.A.	N.A.	N.A.	N.A.
chr17_78712331_78712331_AACGAACT	chr17	78712330	C	CAACGAACT
chr17_78712785_78712785_T	chr17	78712784	C	CT
chr17_78712988_78712988_G	chr17	78712987	C	CG
chr17_78806917_78806917_C	chr17	78806916	G	GC
chr17_79480537_79480537_C	chr17	79480536	G	GC
chr17_79519883_79519883_C	chr17	79519882	G	GC
chr17_80057597_80057597_GTGA	chr17	80057596	G	GGTGA
chr17_80829111_80829111_AG	chr17	80829110	C	CAG
chr17_8762626_8762626_C	chr17	8762625	G	GC
chr17_8844758_8844758_A	chr17	8844757	G	GA
chr18_10928716_10928716_C	N.A.	N.A.	N.A.	N.A.
chr18_10949495_10949495_CT	chr18	10949494	A	ACT
chr18_12991920_12991920_TG	chr18	12991919	A	ATG
chr18_13615939_13615939_T	chr18	13615938	G	GT
chr18_23771544_23771544_G	chr18	23771543	A	AG

chr18_29238077_29238077_A	N.A.	N.A.	N.A.	N.A.
chr18_2980799_2980799_C	N.A.	N.A.	N.A.	N.A.
chr18_2998683_2998683_T	chr18	2998682	A	AT
chr18_32630114_32630114_T	N.A.	N.A.	N.A.	N.A.
chr18_43267817_43267817_GTGCT	chr18	43267816	G	GGTGCT
chr18_4640412_4640412_A	chr18	4640411	C	CA
chr18_46458351_46458351_T	chr18	46458350	C	CT
chr18_47016722_47016722_AT	chr18	47016721	A	AAT
chr18_51673806_51673806_T	N.A.	N.A.	N.A.	N.A.
chr1_85435396_85435396_A	chr1	85435395	T	TA
chr1_85458443_85458443_C	chr1	85458442	A	AC
chr1_85459300_85459300_AA	N.A.	N.A.	N.A.	N.A.
chr18_55297286_55297286_G	chr18	55297285	T	TG
chr18_56231356_56231356_T	N.A.	N.A.	N.A.	N.A.
chr18_56231792_56231792_T	N.A.	N.A.	N.A.	N.A.
chr18_56808380_56808380_T	chr18	56808379	C	CT
chr18_60173623_60173623_T	N.A.	N.A.	N.A.	N.A.
chr18_60192578_60192578_GGA	chr18	60192577	G	GGGA
chr18_60192584_60192584_GGC	N.A.	N.A.	N.A.	N.A.
chr18_60192742_60192742_TA	chr18	60192741	G	GTA
chr18_60192744_60192744_TC	N.A.	N.A.	N.A.	N.A.
chr18_60708464_60708464_AAAG	chr18	60708463	A	AAAAG
chr18_657276_657276_C	chr18	657275	G	GC
chr18_67624587_67624587_T	chr18	67624586	C	CT
chr18_67826146_67826146_T	chr18	67826145	A	AT
chr18_72920739_72920739_GCGGC	chr18	72920738	A	AGCGGC
chr18_74197006_74197006_TG	chr18	74197005	C	CTG
chr18_74197873_74197873_AAT	chr18	74197872	A	AAAT
chr18_74837845_74837845_A	chr18	74837844	C	CA
chr18_77439128_77439128_T	chr18	77439127	A	AT
chr18_8601071_8601071_T	N.A.	N.A.	N.A.	N.A.
chr18_8602132_8602132_G	chr18	8602131	T	TG
chr18_9116358_9116358_CTG	N.A.	N.A.	N.A.	N.A.
chr18_9120672_9120672_TAT	chr18	9120671	G	GTAT
chr1_90158773_90158773_G	N.A.	N.A.	N.A.	N.A.
chr19_1026958_1026958_C	chr19	1026957	T	TC
chr19_10842031_10842031_T	N.A.	N.A.	N.A.	N.A.
chr19_11671278_11671278_GA	N.A.	N.A.	N.A.	N.A.
chr19_14118038_14118038_T	chr19	14118037	C	CT
chr19_14492069_14492069_TGAG	chr19	14492068	C	CTGAG
chr19_14715585_14715585_T	N.A.	N.A.	N.A.	N.A.
chr1_91486373_91486373_AGTC	chr1	91486372	T	TAGTC
chr19_16188098_16188098_G	chr19	16188097	T	TG
chr19_16188521_16188521_A	chr19	16188520	T	TA
chr19_1651799_1651799_C	N.A.	N.A.	N.A.	N.A.
chr19_16658569_16658569_G	N.A.	N.A.	N.A.	N.A.
chr19_17377497_17377497_A	N.A.	N.A.	N.A.	N.A.
chr19_17516401_17516401_GCTGAAAG	chr19	17516400	A	AGCTGAAAG

chr19_17516587_17516587_T	chr19	17516586	C	CT
chr19_17866485_17866485_A	chr19	17866484	T	TA
chr19_17957719_17957719_G	chr19	17957718	A	AG
chr19_18209185_18209185_C	chr19	18209184	T	TC
chr19_19497092_19497092_C	N.A.	N.A.	N.A.	N.A.
chr1_92269097_92269097_T	chr1	92269096	C	CT
chr19_2394598_2394598_TTTATT	chr19	2394597	C	CTTTATT
chr19_2596369_2596369_A	chr19	2596368	T	TA
chr19_2607592_2607592_A	chr19	2607591	G	GA
chr19_2608315_2608315_A	N.A.	N.A.	N.A.	N.A.
chr19_2622072_2622072_T	N.A.	N.A.	N.A.	N.A.
chr19_3180461_3180461_G	N.A.	N.A.	N.A.	N.A.
chr19_32948886_32948886_T	chr19	32948885	A	AT
chr1_93395749_93395749_GGC	chr1	93395748	A	AGGC
chr19_35838860_35838860_A	chr19	35838859	G	GA
chr19_36421694_36421694_A	N.A.	N.A.	N.A.	N.A.
chr1_93810679_93810679_T	chr1	93810678	C	CT
chr19_38397366_38397366_A	N.A.	N.A.	N.A.	N.A.
chr19_38401891_38401891_GT	chr19	38401890	C	CGT
chr19_38453604_38453604_TCTTT	chr19	38453603	C	CTCTTT
chr19_3984982_3984982_T	chr19	3984981	A	AT
chr19_39881016_39881016_AGA	chr19	39881015	T	TAGA
chr19_40193429_40193429_C	N.A.	N.A.	N.A.	N.A.
chr19_40650015_40650015_A	N.A.	N.A.	N.A.	N.A.
chr19_41304596_41304596_TTAC	chr19	41304595	A	ATTAC
chr19_41772526_41772526_G	N.A.	N.A.	N.A.	N.A.
chr19_41833193_41833193_CA	chr19	41833192	G	GCA
chr19_41869488_41869488_A	chr19	41869487	C	CA
chr1_94312483_94312483_C	chr1	94312482	T	TC
chr19_46172397_46172397_G	chr19	46172396	T	TG
chr19_46195052_46195052_C	chr19	46195051	G	GC
chr19_47614901_47614901_A	chr19	47614900	T	TA
chr19_47614906_47614906_G	N.A.	N.A.	N.A.	N.A.
chr19_47616097_47616097_C	N.A.	N.A.	N.A.	N.A.
chr19_48753257_48753257_TAA	N.A.	N.A.	N.A.	N.A.
chr19_49955616_49955616_AACT	chr19	49955615	G	GAACT
chr19_51868894_51868894_CA	chr19	51868893	T	TCA
chr19_52674185_52674185_G	chr19	52674184	A	AG
chr19_54692897_54692897_CTC	chr19	54692896	T	TCTC
chr19_54704061_54704061_C	chr19	54704060	G	GC
chr19_56154878_56154878_G	chr19	56154877	T	TG
chr19_56159797_56159797_G	chr19	56159796	C	CG
chr19_56914910_56914910_A	chr19	56914909	G	GA
chr19_58514096_58514096_C	chr19	58514095	A	AC
chr19_59068570_59068570_AG	N.A.	N.A.	N.A.	N.A.
chr19_6532449_6532449_TGTG	chr19	6532448	T	TTGTG
chr19_6555439_6555439_A	chr19	6555438	T	TA
chr19_6557329_6557329_T	N.A.	N.A.	N.A.	N.A.

chr1_9688944_9688944_C	N.A.	N.A.	N.A.	N.A.
chr19_7764546_7764546_C	chr19	7764545	G	GC
chr1_9777452_9777452_GA	chr1	9777451	G	GGA
chr19_926280_926280_G	chr19	926279	A	AG
chr20_17859988_17859988_A	chr20	17859987	T	TA
chr20_25336120_25336120_C	chr20	25336119	G	GC
chr20_30865967_30865967_C	chr20	30865966	A	AC
chr20_33103448_33103448_CA	chr20	33103447	C	CCA
chr20_43272820_43272820_C	chr20	43272819	G	GC
chr20_43595695_43595695_G	N.A.	N.A.	N.A.	N.A.
chr20_43596336_43596336_G	chr20	43596335	T	TG
chr20_43598167_43598167_TGAA	chr20	43598166	G	GTGAA
chr20_43599531_43599531_TG	chr20	43599530	T	TTG
chr20_44747652_44747652_G	chr20	44747651	T	TG
chr20_44747717_44747717_C	chr20	44747716	A	AC
chr20_47340080_47340080_C	chr20	47340079	A	AC
chr20_47884015_47884015_T	chr20	47884014	G	GT
chr20_49132763_49132763_T	chr20	49132762	A	AT
chr20_49132766_49132766_G	N.A.	N.A.	N.A.	N.A.
chr20_49434670_49434670_A	N.A.	N.A.	N.A.	N.A.
chr20_4954110_4954110_GACTT	chr20	4954109	G	GGACTT
chr20_49546255_49546255_T	N.A.	N.A.	N.A.	N.A.
chr20_49547015_49547015_G	chr20	49547014	A	AG
chr20_52357251_52357251_A	chr20	52357250	G	GA
chr20_52533910_52533910_T	N.A.	N.A.	N.A.	N.A.
chr20_52540119_52540119_TT	chr20	52540118	C	CTT
chr20_5591398_5591398_C	chr20	5591397	G	GC
chr20_57465565_57465565_C	N.A.	N.A.	N.A.	N.A.
chr20_57582505_57582505_A	chr20	57582504	C	CA
chr20_60914674_60914674_T	chr20	60914673	G	GT
chr20_61550177_61550177_T	chr20	61550176	G	GT
chr20_62362699_62362699_TGG	chr20	62362698	T	TTGG
chr20_62363072_62363072_TGTG	chr20	62363071	T	TTGTG
chr20_62643925_62643925_CGG	N.A.	N.A.	N.A.	N.A.
chr2_101011352_101011352_T	N.A.	N.A.	N.A.	N.A.
chr2_105953939_105953939_C	chr2	105953938	G	GC
chr2_106438636_106438636_C	chr2	106438635	T	TC
chr2_109834611_109834611_A	N.A.	N.A.	N.A.	N.A.
chr2_113414326_113414326_A	chr2	113414325	T	TA
chr2_114196109_114196109_T	N.A.	N.A.	N.A.	N.A.
chr2_114451163_114451163_T	chr2	114451162	A	AT
chr2_114649193_114649193_T	N.A.	N.A.	N.A.	N.A.
chr21_15962587_15962587_TT	chr21	15962586	A	ATT
chr2_121044557_121044557_A	chr2	121044556	C	CA
chr21_26793981_26793981_C	chr21	26793980	G	GC
chr21_26863877_26863877_C	chr21	26863876	A	AC
chr21_26939893_26939893_GAG	chr21	26939892	T	TGAG
chr21_27108076_27108076_G	chr21	27108075	A	AG

chr2_127416703_127416703_T	chr2	127416702	A	AT
chr2_128146148_128146148_AA	chr2	128146147	C	CAA
chr2_131135031_131135031_G	N.A.	N.A.	N.A.	N.A.
chr21_34587537_34587537_G	chr21	34587536	T	TG
chr21_34603311_34603311_T	N.A.	N.A.	N.A.	N.A.
chr21_34610986_34610986_T	chr21	34610985	G	GT
chr21_34752438_34752438_C	chr21	34752437	G	GC
chr21_34915850_34915850_C	chr21	34915849	T	TC
chr2_134989654_134989654_CTC	chr2	134989653	A	ACTC
chr2_134992561_134992561_T	chr2	134992560	A	AT
chr2_134992561_134992561_TT	N.A.	N.A.	N.A.	N.A.
chr2_135043687_135043687_A	chr2	135043686	T	TA
chr21_35321845_35321845_G	chr21	35321844	A	AG
chr2_135339205_135339205_G	chr2	135339204	C	CG
chr21_35348657_35348657_T	N.A.	N.A.	N.A.	N.A.
chr21_36068804_36068804_T	chr21	36068803	A	AT
chr21_38445841_38445841_G	chr21	38445840	T	TG
chr21_38580178_38580178_C	chr21	38580177	G	GC
chr21_38639267_38639267_G	chr21	38639266	A	AG
chr21_38640492_38640492_C	chr21	38640491	G	GC
chr21_38739659_38739659_G	chr21	38739658	C	CG
chr21_38786548_38786548_T	chr21	38786547	G	GT
chr21_40138678_40138678_A	chr21	40138677	C	CA
chr21_42798772_42798772_C	chr21	42798771	T	TC
chr21_42799641_42799641_C	chr21	42799640	A	AC
chr21_42800314_42800314_T	chr21	42800313	A	AT
chr21_43812670_43812670_ATGA	chr21	43812669	G	GATGA
chr21_43944008_43944008_T	chr21	43944007	C	CT
chr21_44584552_44584552_T	chr21	44584551	G	GT
chr21_44859657_44859657_TTTCT	chr21	44859656	G	GTTTCT
chr2_145089750_145089750_G	chr2	145089749	T	TG
chr21_45577431_45577431_A	chr21	45577430	C	CA
chr21_45578264_45578264_T	chr21	45578263	C	CT
chr21_45579882_45579882_AT	chr21	45579881	C	CAT
chr21_45632083_45632083_TGAA	N.A.	N.A.	N.A.	N.A.
chr21_47649745_47649745_G	chr21	47649744	A	AG
chr21_47744904_47744904_G	chr21	47744903	A	AG
chr21_48029176_48029176_T	chr21	48029175	C	CT
chr21_48029177_48029177_G	N.A.	N.A.	N.A.	N.A.
chr2_160753987_160753987_A	chr2	160753986	G	GA
chr2_169325408_169325408_G	chr2	169325407	T	TG
chr2_172182151_172182151_T	chr2	172182150	G	GT
chr2_172380244_172380244_CA	chr2	172380243	T	TCA
chr2_175470585_175470585_T	N.A.	N.A.	N.A.	N.A.
chr2_177862363_177862363_C	chr2	177862362	A	AC
chr2_178029679_178029679_A	N.A.	N.A.	N.A.	N.A.
chr2_182324043_182324043_T	chr2	182324042	G	GT
chr2_187351345_187351345_TG	chr2	187351344	C	CTG

chr2_190539927_190539927_T	chr2	190539926	A	AT
chr2_190817844_190817844_A	chr2	190817843	T	TA
chr2_191396706_191396706_A	chr2	191396705	G	GA
chr2_191398501_191398501_A	chr2	191398500	G	GA
chr2_191398784_191398784_CAGC	chr2	191398783	A	ACAGC
chr2_191558585_191558585_AAAT	chr2	191558584	A	AAAAT
chr2_196515029_196515029_A	chr2	196515028	G	GA
chr2_197133135_197133135_A	chr2	197133134	T	TA
chr2_197280336_197280336_C	chr2	197280335	T	TC
chr2_198128683_198128683_G	chr2	198128682	A	AG
chr2_198172276_198172276_A	N.A.	N.A.	N.A.	N.A.
chr2_198172276_198172276_AA	N.A.	N.A.	N.A.	N.A.
chr2_201727661_201727661_A	chr2	201727660	C	CA
chr2_201997143_201997143_G	chr2	201997142	A	AG
chr2_202016286_202016286_CTC	chr2	202016285	T	TCTC
chr2_203883171_203883171_C	chr2	203883170	A	AC
chr2_207024887_207024887_C	chr2	207024886	G	GC
chr2_208031664_208031664_CGCCGC	chr2	208031663	G	GCGCCGC
chr2_209180235_209180235_TTTG	chr2	209180234	C	CTTTG
chr2_211341205_211341205_C	chr2	211341204	G	GC
chr2_213887222_213887222_T	chr2	213887221	G	GT
chr2_214015171_214015171_G	chr2	214015170	A	AG
chr2_214017669_214017669_T	N.A.	N.A.	N.A.	N.A.
chr2_215672462_215672462_GAGGCTCA	chr2	215672461	T	TGAGGCTCA
chr2_215673219_215673219_A	chr2	215673218	G	GA
chr2_216002611_216002611_T	N.A.	N.A.	N.A.	N.A.
chr2_216980534_216980534_T	chr2	216980533	A	AT
chr2_217088307_217088307_TTG	chr2	217088306	C	CTTG
chr2_217088441_217088441_A	chr2	217088440	T	TA
chr22_17723790_17723790_A	chr22	17723789	G	GA
chr2_218084035_218084035_G	chr2	218084034	A	AG
chr2_219084564_219084564_A	chr2	219084563	T	TA
chr2_219265137_219265137_C	chr2	219265136	T	TC
chr2_220042035_220042035_TCTG	chr2	220042034	C	CTCTG
chr2_220042272_220042272_T	N.A.	N.A.	N.A.	N.A.
chr22_23270540_23270540_A	chr22	23270539	C	CA
chr22_23281553_23281553_CAC	chr22	23281552	A	ACAC
chr22_24141469_24141469_T	N.A.	N.A.	N.A.	N.A.
chr22_24235751_24235751_CATT	N.A.	N.A.	N.A.	N.A.
chr22_24372195_24372195_T	chr22	24372194	C	CT
chr2_224761061_224761061_A	chr2	224761060	T	TA
chr2_225848226_225848226_A	chr2	225848225	G	GA
chr22_27006021_27006021_G	chr22	27006020	A	AG
chr22_29598197_29598197_C	chr22	29598196	G	GC
chr22_29598236_29598236_C	chr22	29598235	G	GC
chr2_231069737_231069737_T	chr2	231069736	C	CT
chr2_231280643_231280643_T	chr2	231280642	A	AT
chr2_231282535_231282535_T	chr2	231282534	C	CT

chr2_231480526_231480526_T	chr2	231480525	A	AT
chr22_31609351_31609351_C	chr22	31609350	A	AC
chr22_32057379_32057379_ACA	chr22	32057378	T	TACA
chr22_32150039_32150039_G	chr22	32150038	A	AG
chr2_232419424_232419424_T	chr2	232419423	C	CT
chr2_232539890_232539890_G	chr2	232539889	C	CG
chr2_232646362_232646362_TCC	N.A.	N.A.	N.A.	N.A.
chr2_233951378_233951378_G	chr2	233951377	T	TG
chr22_34238407_34238407_TA	chr22	34238406	T	TTA
chr2_234294562_234294562_TC	chr2	234294561	T	TTC
chr22_36725328_36725328_A	chr22	36725327	C	CA
chr22_37551811_37551811_G	chr22	37551810	A	AG
chr22_37614704_37614704_ACA	chr22	37614703	G	GACA
chr22_37615447_37615447_ATTG	chr22	37615446	T	TATTG
chr22_37620230_37620230_TGTC	chr22	37620229	T	TTGTC
chr22_37679228_37679228_AG	chr22	37679227	T	TAG
chr22_38036372_38036372_TC	chr22	38036371	T	TTC
chr22_38453649_38453649_G	chr22	38453648	A	AG
chr2_238604859_238604859_TTTG	chr2	238604858	A	ATTTG
chr2_238606496_238606496_TCA	chr2	238606495	G	GTCA
chr2_238661249_238661249_T	chr2	238661248	C	CT
chr22_38793331_38793331_C	chr22	38793330	G	GC
chr22_39464334_39464334_A	chr22	39464333	T	TA
chr22_39493935_39493935_T	chr22	39493934	G	GT
chr22_39714304_39714304_A	N.A.	N.A.	N.A.	N.A.
chr22_41348719_41348719_CAAA	chr22	41348718	T	TCAAA
chr2_24150450_24150450_A	chr2	24150449	T	TA
chr2_241523161_241523161_AAGT	chr2	241523160	G	GAAGT
chr22_42258400_42258400_A	N.A.	N.A.	N.A.	N.A.
chr22_42320196_42320196_A	chr22	42320195	T	TA
chr22_43774153_43774153_AG	chr22	43774152	C	CAG
chr22_44463995_44463995_A	chr22	44463994	G	GA
chr22_46172386_46172386_T	N.A.	N.A.	N.A.	N.A.
chr22_47009752_47009752_TCG	chr22	47009751	C	CTCG
chr22_47396733_47396733_A	N.A.	N.A.	N.A.	N.A.
chr22_48492314_48492314_G	chr22	48492313	A	AG
chr22_48493458_48493458_G	chr22	48493457	A	AG
chr22_50273783_50273783_T	chr22	50273782	G	GT
chr22_50971307_50971307_A	chr22	50971306	C	CA
chr22_50978349_50978349_T	N.A.	N.A.	N.A.	N.A.
chr22_50978654_50978654_A	N.A.	N.A.	N.A.	N.A.
chr22_50979104_50979104_A	chr22	50979103	C	CA
chr2_25625423_25625423_TC	chr2	25625422	G	GTC
chr2_25625427_25625427_TA	N.A.	N.A.	N.A.	N.A.
chr2_25643283_25643283_A	chr2	25643282	T	TA
chr2_26100423_26100423_A	chr2	26100422	G	GA
chr2_26291419_26291419_T	N.A.	N.A.	N.A.	N.A.
chr2_26522787_26522787_A	chr2	26522786	G	GA

chr2_27652397_27652397_GAG	chr2	27652396	C	CGAG
chr2_28976082_28976082_C	chr2	28976081	T	TC
chr2_32583059_32583059_G	chr2	32583058	A	AG
chr2_33141310_33141310_C	N.A.	N.A.	N.A.	N.A.
chr2_33141630_33141630_C	N.A.	N.A.	N.A.	N.A.
chr2_33701998_33701998_G	chr2	33701997	C	CG
chr2_33718673_33718673_A	chr2	33718672	G	GA
chr2_33724831_33724831_A	chr2	33724830	C	CA
chr2_37801530_37801530_G	chr2	37801529	A	AG
chr2_38152940_38152940_C	chr2	38152939	G	GC
chr2_42587859_42587859_AAAG	chr2	42587858	C	CAAAG
chr2_43022084_43022084_TTCT	chr2	43022083	G	GTTCT
chr2_44843676_44843676_T	chr2	44843675	A	AT
chr2_46136550_46136550_T	chr2	46136549	C	CT
chr2_46761908_46761908_CTTA	chr2	46761907	C	CCTTA
chr2_47209517_47209517_N	N.A.	N.A.	N.A.	N.A.
chr2_48544091_48544091_TAG	chr2	48544090	A	ATAG
chr2_54788580_54788580_TT	chr2	54788579	G	GTT
chr2_55275645_55275645_G	N.A.	N.A.	N.A.	N.A.
chr2_55277954_55277954_G	chr2	55277953	T	TG
chr2_55844887_55844887_C	chr2	55844886	A	AC
chr2_61991058_61991058_G	chr2	61991057	C	CG
chr2_62342088_62342088_T	N.A.	N.A.	N.A.	N.A.
chr2_62536478_62536478_C	N.A.	N.A.	N.A.	N.A.
chr2_62567996_62567996_T	chr2	62567995	A	AT
chr2_64766119_64766119_T	chr2	64766118	A	AT
chr2_64873286_64873286_C	chr2	64873285	A	AC
chr2_64879981_64879981_TAAGA	chr2	64879980	G	GTAAGA
chr2_64879983_64879983_CGATC	N.A.	N.A.	N.A.	N.A.
chr2_65158389_65158389_T	chr2	65158388	A	AT
chr2_65216014_65216014_G	chr2	65216013	C	CG
chr2_65454318_65454318_GAA	chr2	65454317	G	GGAA
chr2_65586558_65586558_A	chr2	65586557	G	GA
chr2_68615439_68615439_G	chr2	68615438	A	AG
chr2_68617176_68617176_AAAG	chr2	68617175	C	CAAAG
chr2_70028537_70028537_A	N.A.	N.A.	N.A.	N.A.
chr2_70355575_70355575_T	N.A.	N.A.	N.A.	N.A.
chr2_71294483_71294483_CC	N.A.	N.A.	N.A.	N.A.
chr2_74055615_74055615_A	N.A.	N.A.	N.A.	N.A.
chr2_74408734_74408734_A	chr2	74408733	G	GA
chr2_75660924_75660924_A	chr2	75660923	T	TA
chr2_8441803_8441803_C	chr2	8441802	T	TC
chr2_8453570_8453570_T	chr2	8453569	G	GT
chr2_85153413_85153413_G	chr2	85153412	A	AG
chr2_85171804_85171804_C	chr2	85171803	G	GC
chr2_85645772_85645772_CG	chr2	85645771	A	ACG
chr2_86849177_86849177_A	chr2	86849176	G	GA
chr2_96823989_96823989_C	chr2	96823988	G	GC

chr2_98547414_98547414_C	chr2	98547413	T	TC
chr2_98611063_98611063_T	N.A.	N.A.	N.A.	N.A.
chr2_99076525_99076525_TC	chr2	99076524	T	TTC
chr2_99103118_99103118_AGA	chr2	99103117	C	CAGA
chr2_99954277_99954277_T	chr2	99954276	C	CT
chr3_100321908_100321908_A	chr3	100321907	G	GA
chr3_10068663_10068663_TG	chr3	10068662	C	CTG
chr3_104240207_104240207_T	chr3	104240206	C	CT
chr3_105521560_105521560_ACA	chr3	105521559	T	TACA
chr3_107318773_107318773_GA	chr3	107318772	T	TGA
chr3_111833192_111833192_G	N.A.	N.A.	N.A.	N.A.
chr3_114173815_114173815_AG	N.A.	N.A.	N.A.	N.A.
chr3_119279016_119279016_TGTCA	chr3	119279015	T	TTGTCA
chr3_121813581_121813581_T	chr3	121813580	C	CT
chr3_122400395_122400395_G	N.A.	N.A.	N.A.	N.A.
chr3_125076161_125076161_CACG	chr3	125076160	A	ACACG
chr3_125235853_125235853_TTTAT	chr3	125235852	A	ATTTAT
chr3_127469477_127469477_AC	chr3	127469476	G	GAC
chr3_127493814_127493814_AA	chr3	127493813	C	CAA
chr3_128399201_128399201_GGG	N.A.	N.A.	N.A.	N.A.
chr3_130571838_130571838_T	chr3	130571837	G	GT
chr3_130572893_130572893_GAAGAT	chr3	130572892	A	AGAAGAT
chr3_13060767_13060767_C	chr3	13060766	T	TC
chr3_133167807_133167807_T	chr3	133167806	G	GT
chr3_133167808_133167808_G	N.A.	N.A.	N.A.	N.A.
chr3_133295402_133295402_G	chr3	133295401	A	AG
chr3_133381523_133381523_A	chr3	133381522	C	CA
chr3_13456014_13456014_A	chr3	13456013	C	CA
chr3_134571507_134571507_GT	chr3	134571506	G	GGT
chr3_139108251_139108251_TG	chr3	139108250	C	CTG
chr3_14472596_14472596_AT	chr3	14472595	C	CAT
chr3_14692745_14692745_A	chr3	14692744	T	TA
chr3_15333009_15333009_A	N.A.	N.A.	N.A.	N.A.
chr3_156273171_156273171_A	chr3	156273170	T	TA
chr3_15689782_15689782_AGAA	chr3	15689781	T	TAGAA
chr3_15689783_15689783_GAAT	N.A.	N.A.	N.A.	N.A.
chr3_16420313_16420313_C	chr3	16420312	T	TC
chr3_16970450_16970450_A	chr3	16970449	C	CA
chr3_170074002_170074002_AAT	chr3	170074001	A	AAAT
chr3_170969813_170969813_TACTT	chr3	170969812	C	CTACTT
chr3_171002398_171002398_A	chr3	171002397	T	TA
chr3_171003775_171003775_AGG	chr3	171003774	C	CAGG
chr3_171064518_171064518_A	chr3	171064517	G	GA
chr3_172468194_172468194_AA	N.A.	N.A.	N.A.	N.A.
chr3_176912755_176912755_TT	chr3	176912754	C	CTT
chr3_176914531_176914531_T	chr3	176914530	C	CT
chr3_177075947_177075947_G	chr3	177075946	T	TG
chr3_178865366_178865366_T	N.A.	N.A.	N.A.	N.A.

chr3_178865775_178865775_C	chr3	178865774	G	GC
chr3_180050528_180050528_TT	chr3	180050527	G	GTT
chr3_182876667_182876667_T	chr3	182876666	G	GT
chr3_182876672_182876672_C	N.A.	N.A.	N.A.	N.A.
chr3_185654465_185654465_T	chr3	185654464	G	GT
chr3_186704765_186704765_A	chr3	186704764	T	TA
chr3_186759809_186759809_TA	chr3	186759808	C	CTA
chr3_191018895_191018895_C	chr3	191018894	T	TC
chr3_194207767_194207767_C	chr3	194207766	T	TC
chr3_196168489_196168489_CA	chr3	196168488	C	CCA
chr3_197023834_197023834_C	chr3	197023833	G	GC
chr3_197461282_197461282_A	N.A.	N.A.	N.A.	N.A.
chr3_25706308_25706308_CA	N.A.	N.A.	N.A.	N.A.
chr3_42054465_42054465_AAG	chr3	42054464	A	AAAG
chr3_42055521_42055521_CCGGGC	chr3	42055520	G	GCCGGGC
chr3_42055559_42055559_C	chr3	42055558	G	GC
chr3_44380600_44380600_AA	chr3	44380599	C	CAA
chr3_46996710_46996710_C	chr3	46996709	T	TC
chr3_47845039_47845039_AG	chr3	47845038	C	CAG
chr3_505172_505172_C	chr3	505171	A	AC
chr3_56816700_56816700_TTTGT	chr3	56816699	G	GTTTGT
chr3_59466299_59466299_TGT	chr3	59466298	A	ATGT
chr3_72149653_72149653_A	chr3	72149652	T	TA
chr3_75691143_75691143_G	N.A.	N.A.	N.A.	N.A.
chr3_98246776_98246776_CA	chr3	98246775	T	TCA
chr3_98255953_98255953_C	N.A.	N.A.	N.A.	N.A.
chr3_98255954_98255954_T	N.A.	N.A.	N.A.	N.A.
chr3_98255954_98255954_TT	N.A.	N.A.	N.A.	N.A.
chr4_10098422_10098422_AGA	chr4	10098421	G	GAGA
chr4_10119802_10119802_TTTT	chr4	10119801	C	CTTTT
chr4_106069031_106069031_A	chr4	106069030	G	GA
chr4_114676826_114676826_CCA	chr4	114676825	T	TCCA
chr4_116877728_116877728_TT	chr4	116877727	C	CTT
chr4_1197181_1197181_C	N.A.	N.A.	N.A.	N.A.
chr4_120390760_120390760_A	chr4	120390759	T	TA
chr4_122721714_122721714_G	chr4	122721713	T	TG
chr4_123073940_123073940_T	chr4	123073939	C	CT
chr4_1243280_1243280_GCCGCA	chr4	1243279	C	CGCCGCA
chr4_124903_124903_C	chr4	124902	G	GC
chr4_125479_125479_G	chr4	125478	A	AG
chr4_129596168_129596168_A	chr4	129596167	T	TA
chr4_14857161_14857161_T	chr4	14857160	C	CT
chr4_148720963_148720963_T	N.A.	N.A.	N.A.	N.A.
chr4_148757806_148757806_T	chr4	148757805	C	CT
chr4_152019689_152019689_T	chr4	152019688	C	CT
chr4_153600689_153600689_T	chr4	153600688	G	GT
chr4_154409153_154409153_GAGA	chr4	154409152	T	TGAGA
chr4_15772832_15772832_ATT	chr4	15772831	C	CATT

chr4_160100358_160100358_A	chr4	160100357	C	CA
chr4_184366630_184366630_GCACA	chr4	184366629	T	TGCACA
chr4_185188034_185188034_A	chr4	185188033	C	CA
chr4_185200717_185200717_T	chr4	185200716	A	AT
chr4_185205514_185205514_TTCCT	chr4	185205513	C	CTTCCT
chr4_185205518_185205518_T	N.A.	N.A.	N.A.	N.A.
chr4_185338996_185338996_C	chr4	185338995	A	AC
chr4_185736644_185736644_G	chr4	185736643	T	TG
chr4_186231585_186231585_CT	chr4	186231584	G	GCT
chr4_186231586_186231586_TT	N.A.	N.A.	N.A.	N.A.
chr4_186316245_186316245_T	chr4	186316244	C	CT
chr4_187112348_187112348_GTT	chr4	187112347	C	CGTT
chr4_25876701_25876701_AAGACAA	chr4	25876700	C	CAAGACAA
chr4_2693502_2693502_A	chr4	2693501	T	TA
chr4_2757461_2757461_C	chr4	2757460	T	TC
chr4_2802277_2802277_CC	N.A.	N.A.	N.A.	N.A.
chr4_2814583_2814583_AT	chr4	2814582	C	CAT
chr4_3204576_3204576_A	N.A.	N.A.	N.A.	N.A.
chr4_36325187_36325187_G	chr4	36325186	T	TG
chr4_38080728_38080728_T	chr4	38080727	G	GT
chr4_38136035_38136035_AAGATA	chr4	38136034	T	TAAGATA
chr4_38762352_38762352_G	N.A.	N.A.	N.A.	N.A.
chr4_38807364_38807364_T	chr4	38807363	A	AT
chr4_38807832_38807832_T	chr4	38807831	G	GT
chr4_38807839_38807839_G	N.A.	N.A.	N.A.	N.A.
chr4_38857495_38857495_TAAC	N.A.	N.A.	N.A.	N.A.
chr4_39715169_39715169_T	chr4	39715168	C	CT
chr4_39717472_39717472_TTTG	chr4	39717471	A	ATTTG
chr4_40192463_40192463_T	chr4	40192462	A	AT
chr4_40200549_40200549_T	chr4	40200548	C	CT
chr4_40200553_40200553_G	N.A.	N.A.	N.A.	N.A.
chr4_40579663_40579663_AA	N.A.	N.A.	N.A.	N.A.
chr4_40765421_40765421_TGG	chr4	40765420	T	TTGG
chr4_41992347_41992347_TGA	chr4	41992346	G	GTGA
chr4_42659886_42659886_T	chr4	42659885	C	CT
chr4_47465193_47465193_G	chr4	47465192	A	AG
chr4_53587941_53587941_AGAA	chr4	53587940	T	TAGAA
chr4_54569771_54569771_A	chr4	54569770	T	TA
chr4_57623684_57623684_T	N.A.	N.A.	N.A.	N.A.
chr4_6202954_6202954_CACT	chr4	6202953	A	ACACT
chr4_68566234_68566234_T	N.A.	N.A.	N.A.	N.A.
chr4_701008_701008_A	chr4	701007	C	CA
chr4_83352178_83352178_TT	chr4	83352177	C	CTT
chr4_83931524_83931524_C	chr4	83931523	T	TC
chr4_83934558_83934558_AG	chr4	83934557	A	AAG
chr4_84095566_84095566_T	chr4	84095565	C	CT
chr4_84102494_84102494_A	chr4	84102493	G	GA
chr4_84203115_84203115_T	N.A.	N.A.	N.A.	N.A.

chr4_88140650_88140650_AA	chr4	88140649	C	CAA
chr4_89378057_89378057_G	chr4	89378056	C	CG
chr4_94669859_94669859_TAACTT	chr4	94669858	A	ATAACTT
chr5_100161834_100161834_T	chr5	100161833	A	AT
chr5_100238074_100238074_A	chr5	100238073	G	GA
chr5_10757029_10757029_GT	chr5	10757028	A	AGT
chr5_110564346_110564346_A	chr5	110564345	T	TA
chr5_118668741_118668741_T	chr5	118668740	G	GT
chr5_125759525_125759525_CT	chr5	125759524	A	ACT
chr5_130710944_130710944_AA	chr5	130710943	C	CAA
chr5_131438682_131438682_A	chr5	131438681	T	TA
chr5_131756870_131756870_T	chr5	131756869	A	AT
chr5_131759932_131759932_AAGAAAGA	chr5	131759931	G	GAAGAAAGA
chr5_131795239_131795239_CTT	chr5	131795238	C	CCTT
chr5_131827653_131827653_G	chr5	131827652	A	AG
chr5_133860236_133860236_C	chr5	133860235	A	AC
chr5_139391817_139391817_C	chr5	139391816	A	AC
chr5_14145048_14145048_G	chr5	14145047	A	AG
chr5_143171728_143171728_C	chr5	143171727	A	AC
chr5_14665180_14665180_C	chr5	14665179	T	TC
chr5_149160053_149160053_G	chr5	149160052	T	TG
chr5_149789442_149789442_C	chr5	149789441	A	AC
chr5_1499052_1499052_ACAC	chr5	1499051	T	TACAC
chr5_150538891_150538891_T	N.A.	N.A.	N.A.	N.A.
chr5_156543785_156543785_T	N.A.	N.A.	N.A.	N.A.
chr5_158275320_158275320_A	chr5	158275319	T	TA
chr5_158437494_158437494_C	chr5	158437493	T	TC
chr5_158458480_158458480_CCT	chr5	158458479	G	GCCT
chr5_158479018_158479018_A	chr5	158479017	C	CA
chr5_158633970_158633970_A	chr5	158633969	C	CA
chr5_159893217_159893217_TAGC	chr5	159893216	A	ATAGC
chr5_167655606_167655606_CACT	N.A.	N.A.	N.A.	N.A.
chr5_171592638_171592638_A	chr5	171592637	C	CA
chr5_17257437_17257437_AAAAG	chr5	17257436	A	AAAAAG
chr5_176840085_176840085_C	chr5	176840084	G	GC
chr5_176937910_176937910_T	chr5	176937909	G	GT
chr5_176937912_176937912_A	N.A.	N.A.	N.A.	N.A.
chr5_177543394_177543394_AGAGA	chr5	177543393	C	CAGAGA
chr5_177557822_177557822_C	chr5	177557821	A	AC
chr5_178978839_178978839_A	chr5	178978838	C	CA
chr5_179061889_179061889_C	N.A.	N.A.	N.A.	N.A.
chr5_179950453_179950453_T	chr5	179950452	A	AT
chr5_27473533_27473533_T	chr5	27473532	G	GT
chr5_33848321_33848321_A	N.A.	N.A.	N.A.	N.A.
chr5_43009434_43009434_T	chr5	43009433	A	AT
chr5_5423565_5423565_G	chr5	5423564	C	CG
chr5_55390610_55390610_C	chr5	55390609	T	TC
chr5_61602958_61602958_C	chr5	61602957	T	TC

chr5_61708833_61708833_GA	chr5	61708832	C	CGA
chr5_64069366_64069366_CTT	chr5	64069365	C	CCTT
chr5_64920425_64920425_G	chr5	64920424	C	CG
chr5_65914497_65914497_TTTA	chr5	65914496	G	GTTTA
chr5_68671454_68671454_TC	chr5	68671453	G	GTC
chr5_72860726_72860726_A	chr5	72860725	T	TA
chr5_74820448_74820448_G	N.A.	N.A.	N.A.	N.A.
chr5_75698685_75698685_C	N.A.	N.A.	N.A.	N.A.
chr5_78024772_78024772_AA	chr5	78024771	G	GAA
chr5_78204237_78204237_A	chr5	78204236	G	GA
chr5_88029598_88029598_A	N.A.	N.A.	N.A.	N.A.
chr5_88042439_88042439_T	N.A.	N.A.	N.A.	N.A.
chr5_88058401_88058401_T	chr5	88058400	A	AT
chr5_88123674_88123674_A	N.A.	N.A.	N.A.	N.A.
chr5_94038599_94038599_T	chr5	94038598	A	AT
chr5_95063002_95063002_A	chr5	95063001	G	GA
chr5_95063004_95063004_G	N.A.	N.A.	N.A.	N.A.
chr5_95775039_95775039_A	chr5	95775038	C	CA
chr5_96213128_96213128_A	chr5	96213127	C	CA
chr5_96220667_96220667_G	chr5	96220666	T	TG
chr5_96232251_96232251_AAA	chr5	96232250	G	GAAA
chr5_98364669_98364669_CTAT	chr5	98364668	C	CCTAT
chr6_106971601_106971601_A	chr6	106971600	T	TA
chr6_106973700_106973700_A	chr6	106973699	G	GA
chr6_10723828_10723828_T	N.A.	N.A.	N.A.	N.A.
chr6_107293099_107293099_T	chr6	107293098	A	AT
chr6_110014349_110014349_G	chr6	110014348	T	TG
chr6_111744458_111744458_AAGG	chr6	111744457	A	AAAGG
chr6_112062728_112062728_AAC	chr6	112062727	T	TAAC
chr6_11233376_11233376_CT	chr6	11233375	C	CCT
chr6_11367330_11367330_A	chr6	11367329	T	TA
chr6_11367924_11367924_C	chr6	11367923	T	TC
chr6_11461041_11461041_AC	N.A.	N.A.	N.A.	N.A.
chr6_11461041_11461041_ACAC	N.A.	N.A.	N.A.	N.A.
chr6_11496399_11496399_A	chr6	11496398	G	GA
chr6_11514725_11514725_GA	chr6	11514724	T	TGA
chr6_116212973_116212973_A	N.A.	N.A.	N.A.	N.A.
chr6_117804865_117804865_T	chr6	117804864	C	CT
chr6_119217557_119217557_AA	chr6	119217556	C	CAA
chr6_130016528_130016528_G	chr6	130016527	T	TG
chr6_130070438_130070438_CA	chr6	130070437	G	GCA
chr6_132819604_132819604_TTTA	chr6	132819603	G	GTTTA
chr6_132820280_132820280_CT	chr6	132820279	C	CCT
chr6_137072289_137072289_T	N.A.	N.A.	N.A.	N.A.
chr6_137105033_137105033_TG	chr6	137105032	T	TTG
chr6_137417399_137417399_T	chr6	137417398	A	AT
chr6_138189837_138189837_A	chr6	138189836	T	TA
chr6_138230146_138230146_GA	chr6	138230145	T	TGA

chr6_139093893_139093893_T	N.A.	N.A.	N.A.	N.A.
chr6_139191742_139191742_TGCAAT	chr6	139191741	A	ATGCAAT
chr6_14118584_14118584_CT	chr6	14118583	A	ACT
chr6_146057475_146057475_T	N.A.	N.A.	N.A.	N.A.
chr6_146134831_146134831_G	chr6	146134830	A	AG
chr6_14875322_14875322_T	N.A.	N.A.	N.A.	N.A.
chr6_14876915_14876915_TC	chr6	14876914	T	TTC
chr6_149377117_149377117_AT	chr6	149377116	C	CAT
chr6_149433937_149433937_A	N.A.	N.A.	N.A.	N.A.
chr6_149637765_149637765_T	chr6	149637764	C	CT
chr6_151086087_151086087_T	chr6	151086086	A	AT
chr6_159224229_159224229_TT	chr6	159224228	C	CTT
chr6_159230258_159230258_ACAG	chr6	159230257	C	CACAG
chr6_159237757_159237757_AT	chr6	159237756	A	AAT
chr6_15990257_15990257_A	chr6	15990256	T	TA
chr6_15990258_15990258_A	N.A.	N.A.	N.A.	N.A.
chr6_160398529_160398529_C	chr6	160398528	T	TC
chr6_16422279_16422279_T	chr6	16422278	G	GT
chr6_16439601_16439601_GGG	N.A.	N.A.	N.A.	N.A.
chr6_16482244_16482244_T	N.A.	N.A.	N.A.	N.A.
chr6_166747446_166747446_C	N.A.	N.A.	N.A.	N.A.
chr6_166755689_166755689_G	chr6	166755688	C	CG
chr6_170892921_170892921_C	N.A.	N.A.	N.A.	N.A.
chr6_20320104_20320104_AC	chr6	20320103	A	AAC
chr6_2232391_2232391_T	chr6	2232390	G	GT
chr6_24667206_24667206_CC	chr6	24667205	T	TCC
chr6_24721402_24721402_G	chr6	24721401	A	AG
chr6_25405276_25405276_TG	chr6	25405275	T	TTG
chr6_25992023_25992023_TATC	chr6	25992022	G	GTATC
chr6_26034362_26034362_A	chr6	26034361	G	GA
chr6_26055406_26055406_TG	chr6	26055405	C	CTG
chr6_26055866_26055866_TC	chr6	26055865	G	GTC
chr6_26172718_26172718_A	N.A.	N.A.	N.A.	N.A.
chr6_26195754_26195754_T	N.A.	N.A.	N.A.	N.A.
chr6_26198065_26198065_CT	chr6	26198064	A	ACT
chr6_26235787_26235787_A	chr6	26235786	T	TA
chr6_26366444_26366444_TCTT	chr6	26366443	C	CTCTT
chr6_26474097_26474097_T	chr6	26474096	G	GT
chr6_26474202_26474202_G	N.A.	N.A.	N.A.	N.A.
chr6_26474204_26474204_A	chr6	26474203	T	TA
chr6_27807941_27807941_A	chr6	27807940	G	GA
chr6_27838519_27838519_T	chr6	27838518	C	CT
chr6_27856669_27856669_C	N.A.	N.A.	N.A.	N.A.
chr6_28367705_28367705_T	chr6	28367704	C	CT
chr6_2859023_2859023_C	chr6	2859022	T	TC
chr6_290487_290487_A	N.A.	N.A.	N.A.	N.A.
chr6_293510_293510_CCACC	chr6	293509	T	TCCACC
chr6_293575_293575_T	N.A.	N.A.	N.A.	N.A.

chr6_294361_294361_A	chr6	294360	G	GA
chr6_29690885_29690885_C	N.A.	N.A.	N.A.	N.A.
chr6_29894637_29894637_A	chr6	29894636	C	CA
chr6_299323_299323_A	N.A.	N.A.	N.A.	N.A.
chr6_29934131_29934131_T	chr6	29934130	G	GT
chr6_30179634_30179634_A	chr6	30179633	G	GA
chr6_30181568_30181568_C	chr6	30181567	T	TC
chr6_30181569_30181569_G	N.A.	N.A.	N.A.	N.A.
chr6_30495843_30495843_TA	chr6	30495842	C	CTA
chr6_3069389_3069389_TGT	chr6	3069388	A	ATGT
chr6_31133578_31133578_AAGCCC	chr6	31133577	A	AAAGCCC
chr6_31540482_31540482_TC	chr6	31540481	G	GTC
chr6_323241_323241_TG	N.A.	N.A.	N.A.	N.A.
chr6_32440678_32440678_C	chr6	32440677	T	TC
chr6_32556967_32556967_G	chr6	32556966	A	AG
chr6_32557832_32557832_G	N.A.	N.A.	N.A.	N.A.
chr6_32570948_32570948_G	chr6	32570947	T	TG
chr6_32572895_32572895_CCAG	N.A.	N.A.	N.A.	N.A.
chr6_32573809_32573809_AAG	chr6	32573808	A	AAG,AAAG
chr6_32573809_32573809_AG	N.A.	N.A.	N.A.	N.A.
chr6_32577158_32577158_CTCT	chr6	32577157	G	GCTCT
chr6_32591532_32591532_C	chr6	32591531	A	AC
chr6_32604050_32604050_A	chr6	32604049	T	TA
chr6_32634040_32634040_T	chr6	32634039	C	CT
chr6_32634043_32634043_C	chr6	32634042	T	TC
chr6_32634565_32634565_T	N.A.	N.A.	N.A.	N.A.
chr6_32636662_32636662_CTT	chr6	32636661	C	CCTT
chr6_32820696_32820696_G	chr6	32820695	A	AG
chr6_32822377_32822377_G	N.A.	N.A.	N.A.	N.A.
chr6_32861493_32861493_A	chr6	32861492	T	TA
chr6_32922623_32922623_A	N.A.	N.A.	N.A.	N.A.
chr6_32997505_32997505_C	chr6	32997504	T	TC
chr6_33025793_33025793_A	chr6	33025792	C	CA
chr6_33039590_33039590_GG	chr6	33039589	A	AGG
chr6_33044157_33044157_TATGCT	chr6	33044156	G	GTATGCT
chr6_33281327_33281327_A	N.A.	N.A.	N.A.	N.A.
chr6_33289932_33289932_GG	chr6	33289931	T	TGG
chr6_33360464_33360464_CTC	chr6	33360463	T	TCTC
chr6_34151197_34151197_AACCCC	chr6	34151196	A	AAACCCC
chr6_34649127_34649127_C	chr6	34649126	G	GC
chr6_36083020_36083020_TG	chr6	36083019	C	CTG
chr6_36350774_36350774_T	chr6	36350773	A	AT
chr6_36853858_36853858_CGC	chr6	36853857	T	TCGC
chr6_37226734_37226734_T	chr6	37226733	C	CT
chr6_4022696_4022696_G	chr6	4022695	T	TG
chr6_41169022_41169022_A	chr6	41169021	C	CA
chr6_42749670_42749670_CC	N.A.	N.A.	N.A.	N.A.
chr6_44835303_44835303_T	chr6	44835302	C	CT

chr6_52169523_52169523_A	N.A.	N.A.	N.A.	N.A.
chr6_64284275_64284275_A	chr6	64284274	G	GA
chr6_6586241_6586241_G	chr6	6586240	C	CG
chr6_6589193_6589193_GA	chr6	6589192	G	GGA
chr6_6591133_6591133_CT	chr6	6591132	A	ACT
chr6_70525587_70525587_T	N.A.	N.A.	N.A.	N.A.
chr6_74182440_74182440_A	chr6	74182439	C	CA
chr6_79944097_79944097_AG	chr6	79944096	C	CAG
chr6_79944098_79944098_GG	N.A.	N.A.	N.A.	N.A.
chr6_90538898_90538898_A	N.A.	N.A.	N.A.	N.A.
chr7_102067673_102067673_A	chr7	102067672	C	CA
chr7_102155939_102155939_C	N.A.	N.A.	N.A.	N.A.
chr7_105702701_105702701_A	chr7	105702700	G	GA
chr7_105924830_105924830_GC	chr7	105924829	G	GGC
chr7_1068076_1068076_G	chr7	1068075	C	CG
chr7_114557875_114557875_T	chr7	114557874	G	GT
chr7_114562834_114562834_TT	chr7	114562833	G	GTT
chr7_114564644_114564644_GCG	N.A.	N.A.	N.A.	N.A.
chr7_116663832_116663832_TA	chr7	116663831	T	TTA
chr7_120044840_120044840_TTTG	chr7	120044839	A	ATTTG
chr7_120052576_120052576_GTAT	chr7	120052575	A	AGTAT
chr7_126300959_126300959_G	chr7	126300958	A	AG
chr7_127291542_127291542_AA	chr7	127291541	T	TAA
chr7_128095200_128095200_AG	chr7	128095199	A	AAG
chr7_128379155_128379155_GGGGCT	N.A.	N.A.	N.A.	N.A.
chr7_128777306_128777306_T	chr7	128777305	G	GT
chr7_128786781_128786781_T	N.A.	N.A.	N.A.	N.A.
chr7_129250836_129250836_A	chr7	129250835	T	TA
chr7_129649637_129649637_T	chr7	129649636	G	GT
chr7_130710804_130710804_A	N.A.	N.A.	N.A.	N.A.
chr7_130903994_130903994_AA	N.A.	N.A.	N.A.	N.A.
chr7_134681253_134681253_A	chr7	134681252	T	TA
chr7_134845488_134845488_A	chr7	134845487	T	TA
chr7_134845488_134845488_AA	N.A.	N.A.	N.A.	N.A.
chr7_134857223_134857223_AAAAA	N.A.	N.A.	N.A.	N.A.
chr7_137584308_137584308_G	chr7	137584307	A	AG
chr7_137667194_137667194_G	chr7	137667193	T	TG
chr7_139180074_139180074_GT	chr7	139180073	G	GGT
chr7_148824390_148824390_AAAT	chr7	148824389	G	GAAAT
chr7_150079225_150079225_G	chr7	150079224	T	TG
chr7_150131388_150131388_T	chr7	150131387	C	CT
chr7_150132096_150132096_T	chr7	150132095	C	CT
chr7_151049003_151049003_AC	N.A.	N.A.	N.A.	N.A.
chr7_155088509_155088509_CT	chr7	155088508	A	ACT
chr7_155534998_155534998_TTTA	chr7	155534997	G	GTTTA
chr7_20233406_20233406_T	chr7	20233405	C	CT
chr7_20258385_20258385_TCA	chr7	20258384	T	TTCA
chr7_20260390_20260390_T	N.A.	N.A.	N.A.	N.A.

chr7_24883997_24883997_T	chr7	24883996	A	AT
chr7_25018909_25018909_A	N.A.	N.A.	N.A.	N.A.
chr7_2933815_2933815_A	N.A.	N.A.	N.A.	N.A.
chr7_32555501_32555501_A	chr7	32555500	G	GA
chr7_35840899_35840899_C	chr7	35840898	G	GC
chr7_37861359_37861359_A	chr7	37861358	G	GA
chr7_42964724_42964724_ACAT	chr7	42964723	C	CACAT
chr7_43624884_43624884_A	chr7	43624883	G	GA
chr7_43775397_43775397_G	N.A.	N.A.	N.A.	N.A.
chr7_43804408_43804408_ATC	chr7	43804407	T	TATC
chr7_45060459_45060459_AG	chr7	45060458	T	TAG
chr7_4785516_4785516_A	chr7	4785515	C	CA
chr7_55601825_55601825_T	N.A.	N.A.	N.A.	N.A.
chr7_5571987_5571987_GCTACA	chr7	5571986	C	CGCTACA
chr7_6120989_6120989_C	chr7	6120988	G	GC
chr7_64499938_64499938_G	chr7	64499937	C	CG
chr7_66118660_66118660_TTTG	chr7	66118659	A	ATTTG
chr7_69268514_69268514_A	chr7	69268513	G	GA
chr7_72090866_72090866_C	chr7	72090865	G	GC
chr7_73507168_73507168_G	N.A.	N.A.	N.A.	N.A.
chr7_74001787_74001787_A	N.A.	N.A.	N.A.	N.A.
chr7_77056356_77056356_ATC	chr7	77056355	T	TATC
chr7_77429319_77429319_T	N.A.	N.A.	N.A.	N.A.
chr7_8168280_8168280_A	chr7	8168279	G	GA
chr7_8184269_8184269_T	chr7	8184268	G	GT
chr7_870229_870229_C	chr7	870228	T	TC
chr7_90338944_90338944_A	chr7	90338943	G	GA
chr7_91569886_91569886_A	chr7	91569885	G	GA
chr7_94285090_94285090_AAA	chr7	94285089	G	GAAA
chr7_962641_962641_CA	chr7	962640	C	CCA
chr7_97500816_97500816_A	chr7	97500815	T	TA
chr7_97601007_97601007_AC	chr7	97601006	T	TAC
chr7_97880624_97880624_A	chr7	97880623	C	CA
chr8_101395142_101395142_AA	chr8	101395141	T	TAA
chr8_101512900_101512900_G	chr8	101512899	A	AG
chr8_103816786_103816786_A	chr8	103816785	T	TA
chr8_10767811_10767811_T	chr8	10767810	G	GT
chr8_11057498_11057498_T	N.A.	N.A.	N.A.	N.A.
chr8_11315593_11315593_AGA	chr8	11315592	C	CAGA
chr8_11348631_11348631_C	chr8	11348630	A	AC
chr8_11352828_11352828_AG	chr8	11352827	C	CAG
chr8_11390612_11390612_T	chr8	11390611	G	GT
chr8_116235032_116235032_A	chr8	116235031	C	CA
chr8_11662600_11662600_T	chr8	11662599	A	AT
chr8_119521170_119521170_T	chr8	119521169	A	AT
chr8_12611338_12611338_T	chr8	12611337	C	CT
chr8_128315380_128315380_GCC	chr8	128315379	A	AGCC
chr8_128569703_128569703_CA	chr8	128569702	C	CCA

chr8_130563130_130563130_C	chr8	130563129	T	TC
chr8_131821224_131821224_T	chr8	131821223	C	CT
chr8_131821836_131821836_G	chr8	131821835	T	TG
chr8_142128044_142128044_A	N.A.	N.A.	N.A.	N.A.
chr8_142128762_142128762_CC	N.A.	N.A.	N.A.	N.A.
chr8_144363736_144363736_G	chr8	144363735	T	TG
chr8_145733795_145733795_G	chr8	145733794	C	CG
chr8_146078597_146078597_T	N.A.	N.A.	N.A.	N.A.
chr8_17781517_17781517_TTTT	chr8	17781516	G	GTTTT
chr8_29386802_29386802_A	chr8	29386801	C	CA
chr8_30515019_30515019_A	N.A.	N.A.	N.A.	N.A.
chr8_42013564_42013564_ATG	chr8	42013563	T	TATG
chr8_52810726_52810726_TGATTTG	chr8	52810725	C	CTGATTTG
chr8_54933895_54933895_A	N.A.	N.A.	N.A.	N.A.
chr8_54983980_54983980_AT	chr8	54983979	A	AAT
chr8_54983980_54983980_GT	N.A.	N.A.	N.A.	N.A.
chr8_59233976_59233976_A	N.A.	N.A.	N.A.	N.A.
chr8_6270792_6270792_ATC	chr8	6270791	T	TATC
chr8_6275042_6275042_T	N.A.	N.A.	N.A.	N.A.
chr8_6605724_6605724_T	N.A.	N.A.	N.A.	N.A.
chr8_6605777_6605777_T	N.A.	N.A.	N.A.	N.A.
chr8_72750367_72750367_T	N.A.	N.A.	N.A.	N.A.
chr8_74885158_74885158_C	chr8	74885157	A	AC
chr8_77911194_77911194_T	N.A.	N.A.	N.A.	N.A.
chr8_81009376_81009376_A	chr8	81009375	T	TA
chr8_81016693_81016693_AC	chr8	81016692	A	AAC
chr8_8499196_8499196_A	N.A.	N.A.	N.A.	N.A.
chr8_91640864_91640864_GT	chr8	91640863	A	AGT
chr8_95447938_95447938_A	chr8	95447937	T	TA
chr8_96102379_96102379_GT	chr8	96102378	G	GGT
chr9_100748391_100748391_C	N.A.	N.A.	N.A.	N.A.
chr9_100797376_100797376_T	N.A.	N.A.	N.A.	N.A.
chr9_103114305_103114305_CTG	chr9	103114304	T	TCTG
chr9_106856105_106856105_C	chr9	106856104	A	AC
chr9_106856146_106856146_G	chr9	106856145	A	AG
chr9_114789697_114789697_T	N.A.	N.A.	N.A.	N.A.
chr9_114790741_114790741_CA	chr9	114790740	C	CCA
chr9_114793678_114793678_AA	chr9	114793677	C	CAA
chr9_123696576_123696576_T	N.A.	N.A.	N.A.	N.A.
chr9_123834148_123834148_AAG	chr9	123834147	A	AAAG
chr9_123919036_123919036_A	chr9	123919035	T	TA
chr9_126901881_126901881_TT	N.A.	N.A.	N.A.	N.A.
chr9_127356429_127356429_A	N.A.	N.A.	N.A.	N.A.
chr9_127356429_127356429_T	chr9	127356428	C	CT
chr9_127630578_127630578_A	chr9	127630577	T	TA
chr9_127952431_127952431_CTCT	chr9	127952430	A	ACTCT
chr9_130548590_130548590_C	chr9	130548589	G	GC
chr9_130666113_130666113_G	chr9	130666112	A	AG

chr9_130834006_130834006_T	N.A.	N.A.	N.A.	N.A.
chr9_132403950_132403950_GCCCTCGG	chr9	132403949	C	CGCCCTCGG
chr9_132750962_132750962_A	N.A.	N.A.	N.A.	N.A.
chr9_132816399_132816399_C	chr9	132816398	T	TC
chr9_134608078_134608078_GGCAA	chr9	134608077	T	TGGCAA
chr9_135544858_135544858_A	N.A.	N.A.	N.A.	N.A.
chr9_137823466_137823466_ATG	chr9	137823465	C	CATG
chr9_140610597_140610597_GG	N.A.	N.A.	N.A.	N.A.
chr9_140656714_140656714_T	chr9	140656713	A	AT
chr9_19128072_19128072_A	chr9	19128071	T	TA
chr9_19233230_19233230_T	chr9	19233229	A	AT
chr9_2014020_2014020_TTTTAA	chr9	2014019	G	GTTTTAA
chr9_2158296_2158296_T	N.A.	N.A.	N.A.	N.A.
chr9_21803730_21803730_CTTAA	chr9	21803729	T	TCTTAA
chr9_273179_273179_A	chr9	273178	G	GA
chr9_32525795_32525795_AG	chr9	32525794	C	CAG
chr9_32552165_32552165_CTC	chr9	32552164	T	TCTC
chr9_33263590_33263590_A	chr9	33263589	G	GA
chr9_35729070_35729070_CT	chr9	35729069	A	ACT
chr9_37292874_37292874_AAC	chr9	37292873	A	AAAC
chr9_37372474_37372474_G	chr9	37372473	T	TG
chr9_37408681_37408681_A	chr9	37408680	G	GA
chr9_5438937_5438937_G	chr9	5438936	A	AG
chr9_5452922_5452922_G	chr9	5452921	T	TG
chr9_5494581_5494581_T	N.A.	N.A.	N.A.	N.A.
chr9_5630338_5630338_C	chr9	5630337	T	TC
chr9_6412272_6412272_A	chr9	6412271	G	GA
chr9_6684887_6684887_GTTT	chr9	6684886	A	AGTTT
chr9_6716430_6716430_C	chr9	6716429	A	AC
chr9_6750454_6750454_G	N.A.	N.A.	N.A.	N.A.
chr9_68408352_68408352_ATC	N.A.	N.A.	N.A.	N.A.
chr9_71160620_71160620_T	chr9	71160619	C	CT
chr9_71548200_71548200_AAAAT	chr9	71548199	A	AAAAAT
chr9_79054475_79054475_A	chr9	79054474	G	GA
chr9_86594483_86594483_A	N.A.	N.A.	N.A.	N.A.
chr9_86739839_86739839_C	N.A.	N.A.	N.A.	N.A.
chr9_93590263_93590263_T	chr9	93590262	A	AT
chr9_95525986_95525986_CT	chr9	95525985	A	ACT
chr9_97583448_97583448_T	chr9	97583447	C	CT
chrX_11778784_11778784_CTT	chrX	11778783	C	CCTT
chrX_12862816_12862816_T	chrX	12862815	G	GT
chrX_12969808_12969808_A	N.A.	N.A.	N.A.	N.A.
chrX_12997704_12997704_T	N.A.	N.A.	N.A.	N.A.
chrX_13003138_13003138_A	N.A.	N.A.	N.A.	N.A.
chrX_13094554_13094554_AAATATG	chrX	13094553	C	CAAATATG
chrX_13094559_13094559_T	N.A.	N.A.	N.A.	N.A.
chrX_13708086_13708086_T	N.A.	N.A.	N.A.	N.A.
chrX_13711297_13711297_GTTA	chrX	13711296	T	TGTTA

chrX_138913511_138913511_AA	N.A.	N.A.	N.A.	N.A.
chrX_1510034_1510034_T	N.A.	N.A.	N.A.	N.A.
chrX_1777434_1777434_T	chrX	1777433	G	GT
chrX_2440174_2440174_AA	N.A.	N.A.	N.A.	N.A.
chrX_40429795_40429795_AAG	chrX	40429794	A	AAAG
chrX_44764381_44764381_T	chrX	44764380	A	AT
chrX_53449337_53449337_G	chrX	53449336	A	AG
chrX_75369436_75369436_G	N.A.	N.A.	N.A.	N.A.
chrX_77320610_77320610_T	N.A.	N.A.	N.A.	N.A.
